# The selectivity filter of the mitochondrial protein import machinery

**DOI:** 10.1186/s12915-020-00888-z

**Published:** 2020-10-29

**Authors:** Sebastian Kreimendahl, Jan Schwichtenberg, Kathrin Günnewig, Lukas Brandherm, Joachim Rassow

**Affiliations:** Institute for Biochemistry and Pathobiochemistry, Ruhr-University Bochum, 44780 Bochum, Germany

**Keywords:** Mitochondria, Protein targeting, TOM complex, Tom40, Tom70, Chaperones, Ion channel, Selectivity filter

## Abstract

**Background:**

The uptake of newly synthesized nuclear-encoded mitochondrial proteins from the cytosol is mediated by a complex of mitochondrial outer membrane proteins comprising a central pore-forming component and associated receptor proteins. Distinct fractions of proteins initially bind to the receptor proteins and are subsequently transferred to the pore-forming component for import. The aim of this study was the identification of the decisive elements of this machinery that determine the specific selection of the proteins that should be imported.

**Results:**

We identified the essential internal targeting signal of the members of the mitochondrial metabolite carrier proteins, the largest protein family of the mitochondria, and we investigated the specific recognition of this signal by the protein import machinery at the mitochondrial outer surface. We found that the outer membrane import receptors facilitated the uptake of these proteins, and we identified the corresponding binding site, marked by cysteine C141 in the receptor protein Tom70. However, in tests both in vivo and in vitro, the import receptors were neither necessary nor sufficient for specific recognition of the targeting signals. Although these signals are unrelated to the amino-terminal presequences that mediate the targeting of other mitochondrial preproteins, they were found to resemble presequences in their strict dependence on a content of positively charged residues as a prerequisite of interactions with the import pore.

**Conclusions:**

The general import pore of the mitochondrial outer membrane appears to represent not only the central channel of protein translocation but also to form the decisive general selectivity filter in the uptake of the newly synthesized mitochondrial proteins.

## Background

Mitochondria developed in evolution from independent organisms, but within the eukaryotic cells, their growth and their activity are dependent on an intensive exchange of metabolites with the surrounding cytosol. The transport of most of these metabolites is mediated by a large group of related proteins of the mitochondrial inner membrane, the members of the mitochondrial carrier family, MCF (corresponding to the solute carrier family 25, SLC25). In the human genome, 53 genes encoding proteins of this family were identified; 35 MCF genes were found in the yeast *Saccharomyces cerevisiae* [1–4]. Each of the encoded proteins is specific for a small subset of the metabolites.

Besides metabolites, also proteins are transported across the mitochondrial membranes. Mitochondria contain about 1000 (in yeast) to 1500 (in humans) different proteins [5, 6]. Nearly all mitochondrial proteins are nuclear-encoded and therefore synthesized outside the mitochondria in the cytosol. The identification and the uptake of these proteins is mediated at the mitochondrial surface by the components of the TOM complex (translocase of the outer membrane). The TOM complex contains a central pore-forming β-barrel protein, Tom40, that is associated with several additional membrane proteins, including Tom20, Tom22, and Tom70, that expose hydrophilic domains on the cytosolic side of the membrane [7–10]. Matrix-targeted mitochondrial proteins carry a positively charged presequence at their N-terminus that binds to the hydrophilic domains of Tom20 and Tom22 for subsequent transfer to Tom40 [11–15]. In this case, the positively charged presequence labels the proteins for mitochondrial import [16–21]. However, nearly all proteins that are targeted to other mitochondrial compartments are lacking a presequence. This large fraction, more than 50% of the 1000–1500 different mitochondrial proteins [5], also comprises the MCF carrier proteins. How are these proteins identified by the TOM complex independently of a presequence?

The MCF proteins show only a weak affinity for Tom20 and Tom22 but a substantial affinity for Tom70, suggesting that Tom70 plays an important role in the recognition of this subset of proteins for subsequent import into the mitochondria [12, 22–24]. In absence of Tom70, the import efficiency of carrier proteins is reduced, but a significant protein import is still possible, indicating some capability of mitochondria to identify carrier proteins also independently of Tom70 [23, 25, 26].

What are the specific structures that could be recognized by the mitochondrial outer membrane TOM complex to identify newly synthesized carrier proteins? MCF carrier proteins typically contain three modules of roughly 100 residues, each module containing two membrane-spanning hydrophobic α-helices, the helices are connected by hydrophilic segments [27]. There is clear evidence that mitochondrial targeting of MCF proteins is mediated by internal signals and that these signals are distributed within all three modules of the proteins, but the locations and the decisive structural features of these signals have been enigmatic [24, 28–33]. Some members of the carrier family were also identified in other cellular membranes. Prominent examples are Ugo1, a protein of the mitochondrial outer membrane [34, 35] and the peroxisomal adenine nucleotide transporter Ant1p of yeast [36]. Mitochondrial targeting signals have been investigated for more than 30 years [37]; however, the targeting signals of the MCF proteins and their corresponding binding sites at the mitochondrial surface are still unknown.

In this study, we investigated this targeting system using the ADP/ATP carrier (AAC) of *Neurospora crassa* as an established model protein. We identified a unique type of a bipartite targeting sequence that is both necessary and sufficient to direct the AAC and related proteins to mitochondria and to the outer membrane TOM complex, and we found that the concept of this bipartite signal was also suitable to address the enigmatic sorting of the proteins Ant1p to peroxisomes and of Ugo1 to the mitochondrial outer membrane. It turned out that Tom70 alone was not able to recognize the intrinsic elements of the carrier targeting sequences. On the other hand, the core of the targeting signal was sufficient to mediate an efficient and highly specific mitochondrial targeting even in the absence of Tom70. We therefore suggest to re-evaluate the function of Tom70 in mitochondrial protein import.

## Results

### A bipartite targeting signal in AAC

**Fig. 1:**
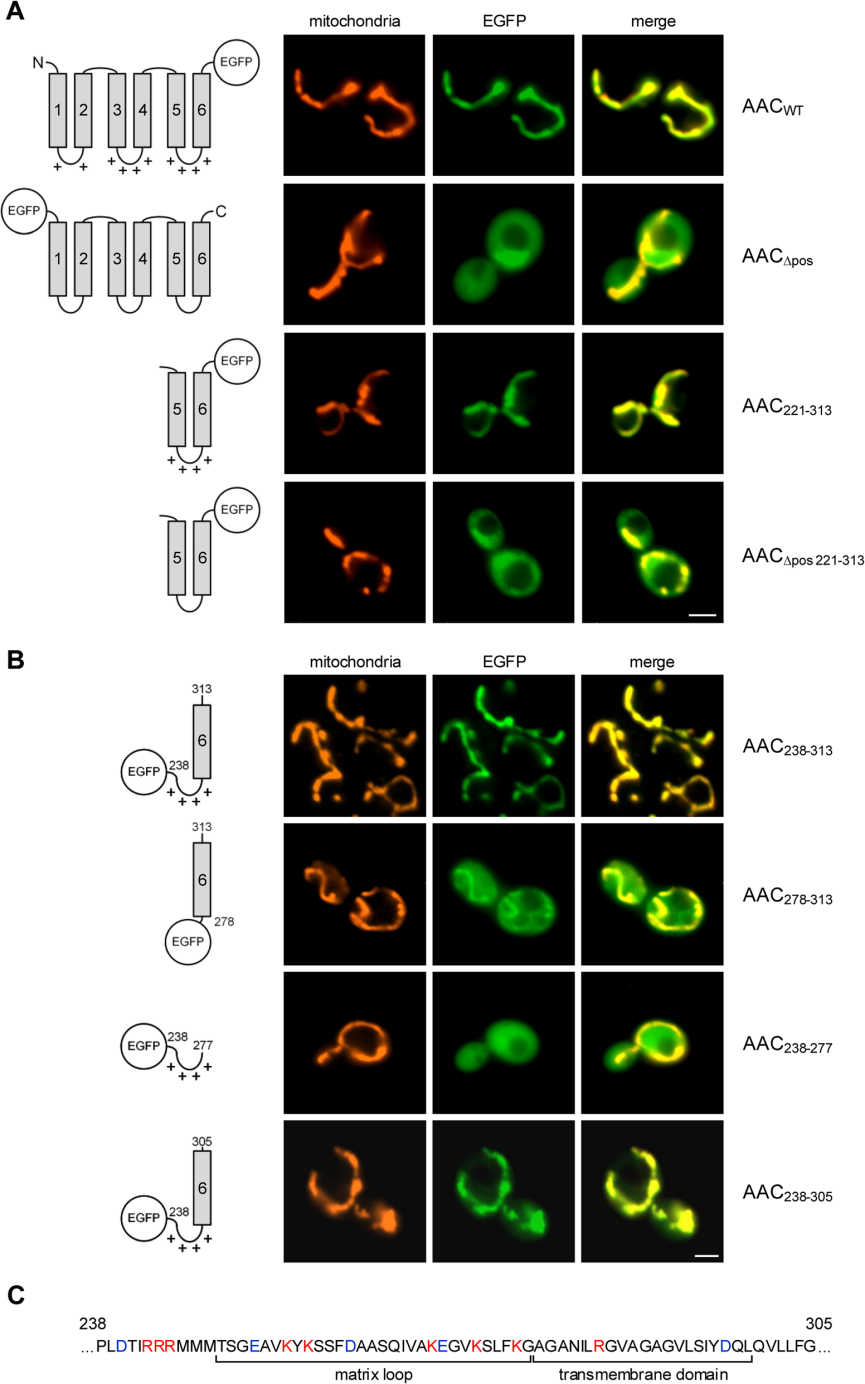
Fluorescence microscopy of yeast cells expressing EGFP-labeled segments of AAC. **a** Upper panel: Images of yeast cells expressing wild type AAC (AAC_WT_, ADP/ATP carrier of *Neurospora crassa*) containing an EGFP moiety at the C-terminus. Second panel: Cells expressing AAC_Δpos_ (all positively charged residues of the matrix loops exchanged against glycine). Third panel: AAC module III (residues 221–313). Lower panel: AAC module III_Δpos_ (the positively charged residues of the matrix loop exchanged against glycine). MitoTracker Orange was used for labelling of the mitochondria. Scale bars, 2 μm. **b** Yeast cells expressing EGFP-labeled segments of AAC module III. **c** Amino acid sequence of *Neurospora crassa* AAC residues 238–305. The assignments are based on the crystal structure of bovine ADP/ATP carrier [1, 40]. Red, positively charged residues; blue, negatively charged residues

To determine the elements of the ADP/ATP carrier (AAC) that are essential for mitochondrial targeting in vivo, we tested a series of hybrid proteins of different parts of the AAC ([38]; UniProtKB – P02723) fused to EGFP (enhanced green fluorescence protein [39]). The constructs were expressed in yeast, and their intracellular distribution was determined by fluorescence microscopy; the mitochondria were labeled using the fluorescent dye MitoTracker Orange (Fig. 1). As expected, intact EGFP-labeled AAC showed a clear mitochondrial localization (Fig. 1a, upper panel).

Transport of the AAC across the mitochondrial outer membrane is membrane potential-independent while subsequent insertion into the mitochondrial inner membrane is essentially driven by the mitochondrial membrane potential [41]. We therefore expected that targeting of the AAC to the mitochondrial outer membrane should be independent of positively charged residues. We tested a construct in which all positively charged amino acids in the three matrix loops of the AAC were substituted by glycine residues (Fig. 1a, second panel). Surprisingly, the construct was distributed in the cytosol, and no labelling of mitochondria was observed. We tested the third module of the AAC separately (residues 221–313) and found that the intact module was efficiently transported to the mitochondria (Fig. 1a, third panel). However, the same module lacking the positively charged residues in the matrix loop had a cytosolic localization (Fig. 1a, lower panel). The positive charges seem to be an essential feature of a module’s targeting signal.

To determine the essential core of the targeting signal, we tested additional constructs (Fig. 1b). A C-terminal part of the AAC (residues 238–313) comprising the third matrix loop and the sixth transmembrane domain still showed a clear mitochondrial location (Fig. 1b, upper panel). A construct lacking the matrix loop was only partially associated with mitochondria (residues 277–313; Fig. 1b, second panel). In many cells, additional structures showed a faint labelling, indicating that the matrix loop is required both for the efficiency and the specificity of targeting. However, the matrix loop alone (residues 238–277) is not an autonomous targeting signal, and the corresponding construct was found exclusively in the cytosol (Fig. 1b, third panel). Eventually, we found that for efficient and specific targeting, a segment containing the matrix loop connected to the sixth transmembrane domain together with additional 6 residues was sufficient (residues 238–305; Fig. 1c).

Strikingly, a construct containing the matrix loop of module III together with the preceding helix 5 remained in the cytosol (Additional file 1: Fig. S1A). To decide if a specific helix is relevant for targeting (helix 5 vs. helix 6) or if a specific orientation is essential (the matrix loop attached to the C- or to the N-terminus of the helix), we substituted helix 5 by helix 6. This construct showed a clear co-localization with mitochondria. On the other hand, helix 6 substituted by helix 5 allowed only some partial co-localization with mitochondria. Hence, both the helix and the orientation matter in efficient targeting: Helix 6 is more efficient in targeting than helix 5 and the helix at the C-terminus of the matrix loop is more relevant for targeting as compared to the N-terminal helix.

An obvious difference between the helices of the AAC is single arginine residues that are specific for the central parts of the even-numbered helices [42]. In module III, this residue corresponds to Arg290 (Additional file 1: Fig. S1A). However, we found that a construct containing a glutamine in this position (Arg290Gln) retained its clear mitochondrial localization. Testing two constructs containing a truncated C-terminal end, it turned out that in this case the constructs quickly lost their targeting function: A polypeptide lacking the amino acids beyond residue 300 showed only partial mitochondrial localization, and loss of additional residues (retaining only 13 hydrophobic residues of helix 6) abolished the targeting function completely. Similarly, a shortening of the matrix loop compromised the targeting function. A construct containing only residues 258–313 showed only a minor co-localization with mitochondria. In summary, the internal targeting sequence of AAC module III appears to correspond to the sequence of the residues 246–305 shown in Fig. 1c.

It is known from many studies that every module of an MCF protein can target the mitochondrial surface independently [24, 29, 43, 44]. Correspondingly, we found that the bipartite segments of matrix loop and transmembrane domain of AAC modules I or II, as well as of yeast dicarboxylate carrier (DIC [45, 46]) module III, showed efficient and specific mitochondrial targeting (Additional file 1: Fig. S1, B and C).

### Recognition of AAC targeting signals at the mitochondrial surface

**Fig. 2:**
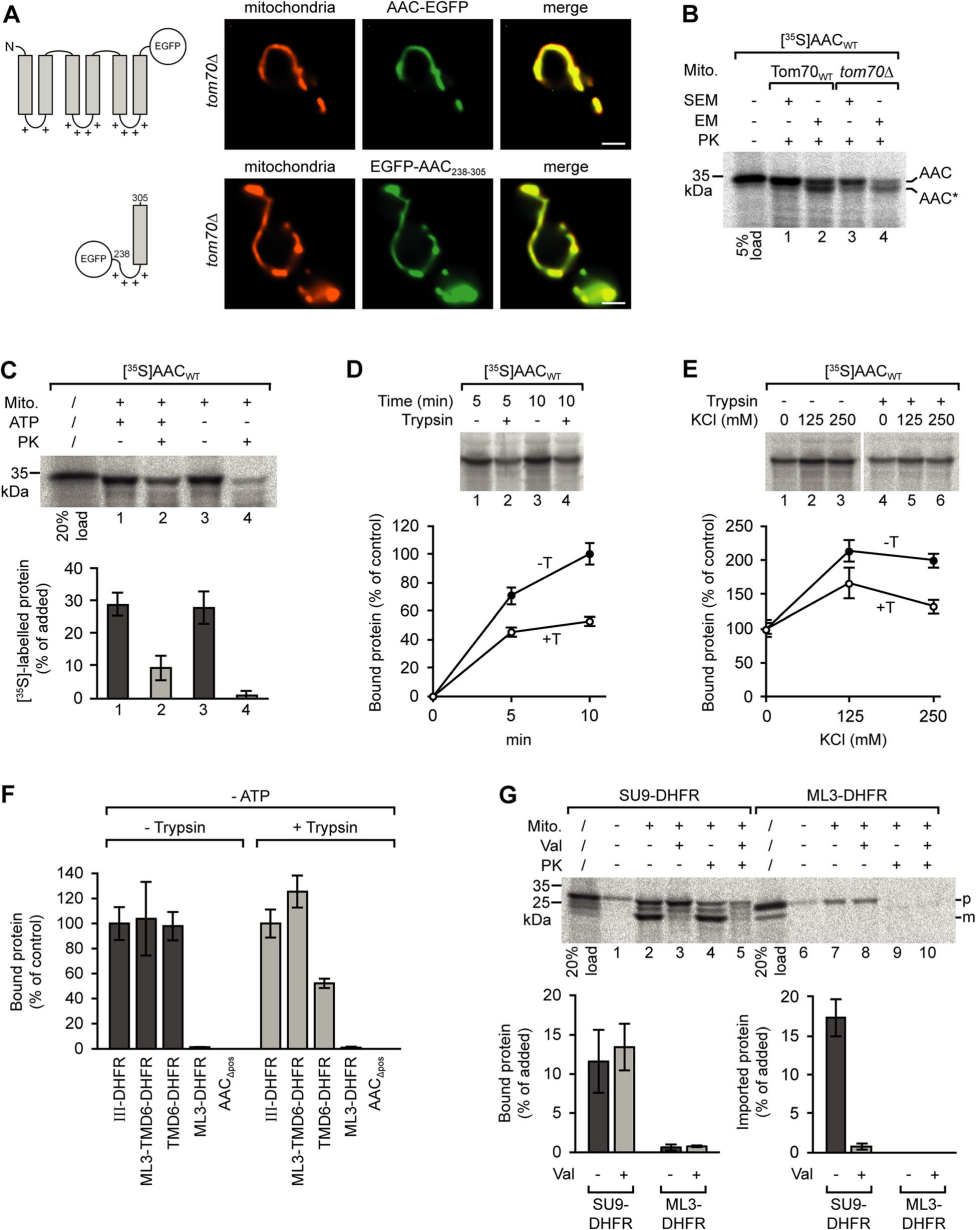
Tom70-independent targeting of AAC to yeast mitochondria. **a** Fluorescence microscopy of cells of a yeast *tom70*Δ strain expressing EGFP-labeled AAC (upper panel) or the EGFP-labeled segment containing the matrix loop of AAC module III and helix 6 (lower panel); scale bars 2 μm. **b** Assembly of the AAC in the mitochondrial inner membrane in WT and *tom70*Δ yeast mitochondria. Isolated mitochondria (30 μg) were incubated with reticulocyte lysate containing [^35^S]-labeled AAC at 25 °C for 10 min. The mitochondria were reisolated and resuspended in isotonic SEM or hypotonic EM buffer for opening of the mitochondrial outer membrane. To determine the amount of imported protein, the samples were incubated in the presence of 75 μg/ml proteinase K on ice for 10 min and the reaction was stopped by addition of 4 mM PMSF. The mitochondria were reisolated and subjected to SDS-PAGE. **c** Binding of [^35^S]-labeled AAC to yeast mitochondria. Isolated mitochondria and reticulocyte lysate containing [^35^S]-labeled AAC were separately depleted of ATP by incubation with apyrase. The mitochondria were subsequently incubated with the reticulocyte lysate (− ATP, samples 2 and 4). In parallel, untreated mitochondria were incubated with [^35^S]-labeled AAC in presence of 2 mM ATP (+ ATP, samples 1 and 2). The mitochondria of samples 3 and 4 were then treated with proteinase K. The mitochondria were reisolated, and the relative amounts of radiolabeled AAC were determined by SDS-PAGE and subsequent analysis using the phosphorimager. Standard deviations (SD), *n* = 4. PK, proteinase K. **d** Binding of [^35^S]-labeled AAC to trypsin-pretreated yeast mitochondria. The trypsin-pretreated mitochondria and reticulocyte lysate containing [^35^S]-labeled AAC were separately incubated with apyrase and subsequently mixed and incubated for 5 or 10 min at 25 °C. Untreated mitochondria (− Trypsin) were used in parallel. The mitochondria were reisolated to determine the relative amounts of bound AAC. The average amount of AAC bound to untreated mitochondria within 10 min was set to 100% (control). SD, *n* ≥ 6. **e** Binding of AAC to mitochondria in the presence of increasing concentrations of KCl. The average amounts of mitochondria-associated AAC obtained in the absence of KCl in the import buffer were set to 100% (control); SD, *n* = 3. **f** Binding of different [^35^S]-labeled polypeptides to trypsin-pretreated vs. untreated yeast mitochondria. The assays were carried out as in **e** using a buffer containing 80 mM KCl. The ATP-depleted reticulocyte lysates contained different segments of the AAC fused to dihydrofolate reductase (DHFR). ML3, matrix loop of AAC module III, residues 238–277; TMD6, transmembrane domain (AAC helix 6); AAC_Δpos_, AAC with all positively charged residues of the matrix loops exchanged against glycine; SD, *n* = 3. **g** Incubation of isolated mitochondria with radiolabeled hybrid proteins containing the presequence (residues 1–69) of subunit 9 of *N. crassa* ATP synthase and DHFR (Su9-DHFR), or the matrix loop of AAC module III and DHFR (ML3-DHFR). SD, *n* = 3. Val, valinomycin; p, precursor protein; m, mature protein

Tom70 is regarded as the major receptor protein for newly synthesized carrier proteins at the mitochondrial outer membrane. However, upon expression of EGFP-labeled AAC in a yeast strain lacking Tom70 (*tom70Δ*), we found that targeting of the AAC to the mitochondria was not compromised (Fig. 2a, upper panel). Correspondingly, also the co-localization of the EGFP-labeled targeting sequence of AAC module III with mitochondria was independent of Tom70 (Fig. 2a, lower panel). The specific mitochondrial localization of both proteins was also retained in the cells of a strain lacking both Tom70 and the related protein Tom71 (data not shown).

The possible role of Tom70 in the uptake of AAC into mitochondria was investigated using an established protease protection assay [47]: AAC was synthesized in reticulocyte lysate in the presence of [^35^S]-labeled methionine and incubated with isolated yeast mitochondria for 10 min at 25 °C, and proteinase K was added to degrade all radiolabeled protein outside the mitochondrial membranes (Fig. 2b). In comparison with wild type mitochondria, uptake of the [^35^S]-labeled AAC into mitochondria isolated form a *tom70* deletion strain was delayed; however, the imported AAC was protected against the externally added protease irrespective of the presence or absence of Tom70 (Fig. 2b, lane 3 vs. lane 1). A proper insertion of AAC into the mitochondrial inner membrane can be verified by protease treatment after opening of the outer membrane. For this purpose, the mitochondria are subjected to an osmotic shock. Under these conditions, a distinct segment of 2–3 kDa of the AAC becomes accessible for the protease [47, 48]. Using this assay, we found that the correct membrane insertion of the AAC was independent of Tom70 (Fig. 2b, lane 4 vs. lane 2).

The involvement of Tom70 in targeting of newly synthesized AAC to the mitochondrial surface was assessed in the experiments shown in Fig. 2c–e. We again synthesized [^35^S]-labeled AAC in reticulocyte lysate, but subsequently depleted the samples of ATP by incubation with apyrase. In parallel, also mitochondria were treated with apyrase. In samples containing both ATP-depleted mitochondria and reticulocyte lysate, only minor amounts of the radiolabeled AAC were imported, most AAC was captured at the mitochondrial surface (Fig. 2c). Using this system, we tested for binding of AAC to mitochondria pretreated with trypsin to remove all receptor sites that were accessible for the protease (Fig. 2d). The trypsin pretreatment of the mitochondria reduced the efficiency of AAC binding by about 50%.

In the next assay, we determined the effects of increasing concentrations of KCl on the efficiency of binding (Fig. 2e). The effects showed a similar pattern with the protease-pretreated and with the untreated mitochondria, with optimal binding at 125 mM KCl. Considering that mitochondrial targeting is mediated both by hydrophilic and by hydrophobic segments of the AAC, it is remarkable that the interactions with the protease-treated and the untreated mitochondria are similarly affected by conditions of different ionic strength.

We then compared the consequences of trypsin pretreatment for binding using different segments of the AAC (Fig. 2f). In these assays, we tested in parallel samples AAC module III, the bipartite targeting signal of module III, helix 6, the intact matrix loop, and full-length AAC lacking the positively charged residues in the matrix loops (AAC_Δpos_). The segments were fused to DHFR (dihydrofolate reductase), a soluble cytosolic protein, to improve the resolution in SDS-PAGE (Additional file 2: Fig. S2A). In Fig. 2f, the amounts of the AAC module III associated with the mitochondria were set to 100%. The different fragments of the AAC bound with different efficiencies to the mitochondria; however, the pattern was similar for the trypsin-pretreated and the untreated mitochondria. Only helix 6 showed a significantly reduced affinity to the trypsin-pretreated organelles.

The results of the assays show that the bipartite targeting signal of the AAC is required for specific binding to mitochondria, but the specificity is not dependent on the import receptor Tom70.

In the presence of ATP, about 30% of radiolabeled AAC that associated with the isolated mitochondria in the import assays was protected against externally added proteinase K (Fig. 2c, lane 1 vs. lane 2). Similar ratios were found with the short construct containing merely the bipartite targeting signal fused to the DHFR (ML3-TMD6-DHFR; Additional file 2: Fig. S2B, lanes 1 and 2 vs. lanes 3 and 4), showing that the construct was not only sufficient for specific transport to the mitochondrial surface but also for insertion into the mitochondrial outer membrane import channel.

It is known from the results of several studies that many mitochondrial preproteins contain internal segments that resemble positively charged presequences of matrix-targeted precursor proteins and are able to mediate mitochondrial targeting [49–52]. However, in direct comparison of the AAC matrix loop with an authentic presequence (of *N. crassa* ATPase subunit 9), the matrix loop was neither able to target a DHFR moiety to mitochondria, nor able to mediate an import into the organelles (Fig. 2g). We conclude that the matrix loop does not act in analogy to a classical presequence.

### Binding of AAC to Tom70

**Fig. 3:**
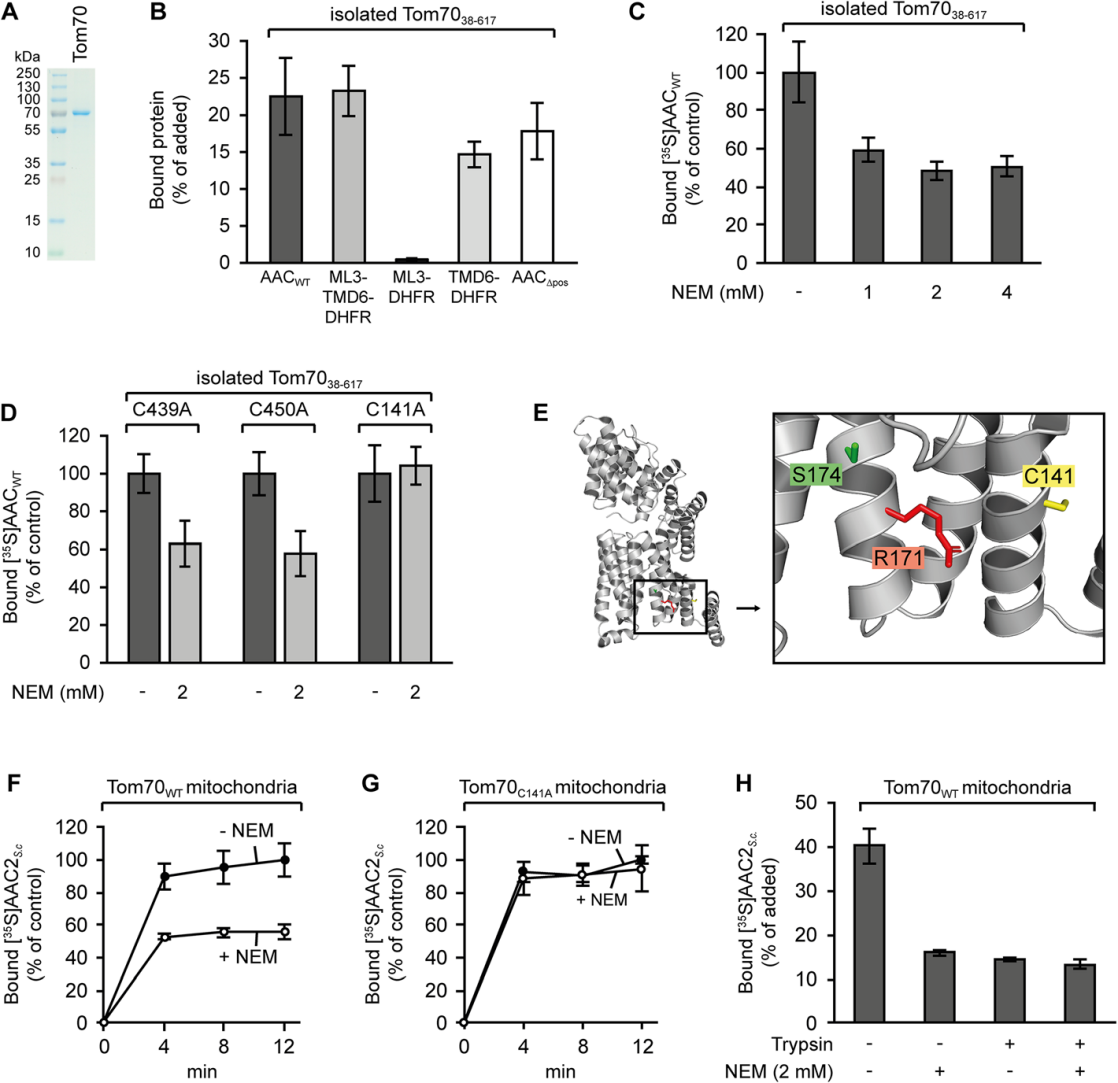
Binding of [^35^S]-labeled polypeptides to the purified cytosolic domain of yeast Tom70 and to isolated mitochondria. **a** SDS-PAGE of 0.75 μg isolated Tom70_cd_ (cytosolic domain, residues 38–617). **b** Binding of [^35^S]-labeled polypeptides to Tom70_cd_. Purified Tom70_cd_ was pre-bound to Ni-NTA beads and incubated with reticulocyte lysate containing radiolabeled wild type AAC (AAC_WT_), or different segments of the AAC fused to DHFR: C-terminal part of AAC module III including the matrix loop and helix 6 (ML3-TMD6, residues 238–313), the matrix loop (ML3, residues 238–277), helix 6 (TMD6, residues 277–313), or the AAC derivative lacking the positively charged residues in the matrix loops (AAC_Δpos_). Bound radiolabeled proteins were quantified using the phosphorimager, SD, *n* = 3. **c** Binding of [^35^S]-labeled AAC_WT_ to isolated Tom70_cd_ pretreated with N-ethylmaleimide (NEM). The assays were performed as in **b**, but the Ni-NTA-bound Tom70_cd_ was preincubated with up to 4 mM NEM as indicated. The amount of bound protein in absence of NEM was set to 100%. SD, *n* = 4. **d** Binding of [^35^S]-labeled AAC_WT_ to mutant versions of Tom70_cd_. Single cysteines in positions 439, 450, or 141, respectively, were exchanged against alanine. The modified Tom70_cd_ proteins were bound to Ni^2+^-NTA, and as indicated, some samples were treated with 2 mM NEM. All beads were washed with assay buffer and subsequently used for binding assays as in **c**. SD, *n* ≥ 6. **e** Location of cysteine residue C141, arginine R171, and serine S174 in Tom70. The structural data were taken from Wu and Sha [53], PDB 2GW1. **f** Binding of [^35^S]-labeled yeast AAC2 to isolated mitochondria containing wild type Tom70. Radiolabeled AAC2 was synthesized in reticulocyte lysate and preincubated with apyrase; the mitochondria were pretreated with 2 mM NEM (+ NEM) or left untreated (− NEM). Binding in absence of NEM within 12 min was set to 100%, SD, *n* = 3. **g** Binding of [^35^S]-labeled AAC2 to mitochondria isolated from a yeast strain expressing Tom70_C141A_. The assay was carried out as in **f**. Binding in absence of NEM within 12 min was set to 100%, SD, *n* = 3. **h** Binding of AAC to mitochondria pretreated with NEM and trypsin, SD, *n* = 3

To investigate the interactions of AAC with isolated Tom70, we expressed the cytosolic domain of yeast Tom70 in *E. coli* (Tom70_cd_; Fig. 3a), bound the purified protein to Ni-NTA agarose beads, and tested for binding of [^35^S]-labeled preproteins following an established protocol [12]. Using this assay, we found about 20–25% of the total amount of radiolabeled AAC associated with Tom70_cd_ (Fig. 3b). Similar ratios were observed with the construct containing the bipartite signal sequence of module III. Negligible amounts were found with the construct containing only the matrix loop (< 1%), but high amounts were also observed with helix 6 alone. The pattern obtained with the different constructs basically resembled the data on the binding to isolated mitochondria (Fig. 2f).

A striking difference was observed with the AAC lacking the positive charges in the matrix loops (AAC_Δpos_). This construct had not shown an association with mitochondria in any of our previous experiments, but in this assay, about 15–20% of the construct bound to the isolated Tom70_cd_. Due to its inability to recognize the positively charged matrix loops, isolated Tom70 seems to be unable to discriminate between the intact AAC and the modified AAC_Δpos_.

Yeast Tom70 is a protein of 617 amino acids containing an N-terminal membrane anchor [54]. The crystal structure of the cytosolic part of yeast Tom70 [53] shows 26 tightly packed α-helices organized in an N-terminal domain (residues 38–222, including a binding site for chaperone proteins in a region near an arginine residue in position 171 [55, 56]) and a C-terminal domain (residues 249–617, containing an array of residues, Glu473, Glu542, and Glu577, suggested to participate in the formation of a specific binding site for targeting signals of mitochondrial precursor proteins [53]). The structure of Tom70 has some flexibility and can adopt at least two different configurations [57, 58]. To determine the domain involved in binding of the AAC, we screened the structure of yeast Tom70 for residues that could be used to block potential interaction sites. We identified three cysteine residues (in positions 141, 439, and 450), and we found that a pretreatment of isolated Tom70_cd_ with NEM (N-ethylmaleimide) caused a reduction of the binding efficiency for wild type AAC by about 50% (Fig. 3c). We separately exchanged the individual cysteine residues against alanine and found that NEM pretreatment inhibited binding of AAC to Tom70_C439A_ and Tom70_C450A_, but binding of AAC to the C141A derivative was no longer affected by the NEM (Fig. 3d). The cysteine C141 of the N-terminal domain of Tom70 seems to be located in the binding site for the AAC while the two cysteine residues 439 and 450 of the C-terminal domain of Tom70 are not involved. This assumption is supported by data of a previous study showing that a residue in the vicinity of cysteine C141, arginine R171 (Fig. 3e), is located in a binding site for Hsp70-bound MCF proteins [53]. We conclude that under the conditions of our experiments, the AAC preferentially binds to this site.

To evaluate the effects of the assay, we pretreated isolated yeast mitochondria with NEM and tested for binding of *S. cerevisiae* AAC2 (Fig. 3f). In direct comparison to untreated mitochondria, the efficiency of AAC2 binding to mitochondria was reduced by the NEM pretreatment by about 50%. With mitochondria containing the modified receptor Tom70_C141A_, the NEM treatment had no effect (Fig. 3g), demonstrating that the inhibitory effect of NEM was dependent on a reaction with the cysteine C141.

Eventually, we compared the effect of NEM on binding of AAC to intact mitochondria and to trypsin-pretreated mitochondria (Fig. 3h). In this assay, no NEM effect was observed with the trypsin-pretreated mitochondria. The amounts of radiolabeled AAC retained with NEM-pretreated mitochondria were similar to the amounts determined for trypsin-pretreated mitochondria, indicating that mitochondrial Tom70 can be completely inactivated by blocking the side chain of cysteine 141.

Remarkably, the pattern of affinities of isolated Tom70 (Fig. 3b–d) differs from the pattern affinities of Tom70 embedded in the TOM complex of the mitochondrial outer membrane (Fig. 3f–h): (1) Isolated Tom70 retains a considerable binding capacity for the AAC after blocking of cysteine C141, but in the outer membrane, inactivation of the same cysteine entails a complete loss of Tom70-dependent binding. (2) Isolated Tom70 binds the AAC lacking the positively charged matrix loops (Fig. 3b), but at the outer membrane, no significant binding of this protein is detectable (Fig. 2f). Tom70-mediated binding of AAC to mitochondria involves an ability to recognize the positively charged AAC matrix loops. However, this function depends on structures that are even retained if the mitochondria are pretreated with trypsin (Fig. 2f). Tom70 seems to require a cooperation with structures beyond the receptor stage to ensure the specificity of mitochondrial targeting.

### Transfer of AAC from Tom70 to the import channel

**Fig. 4:**
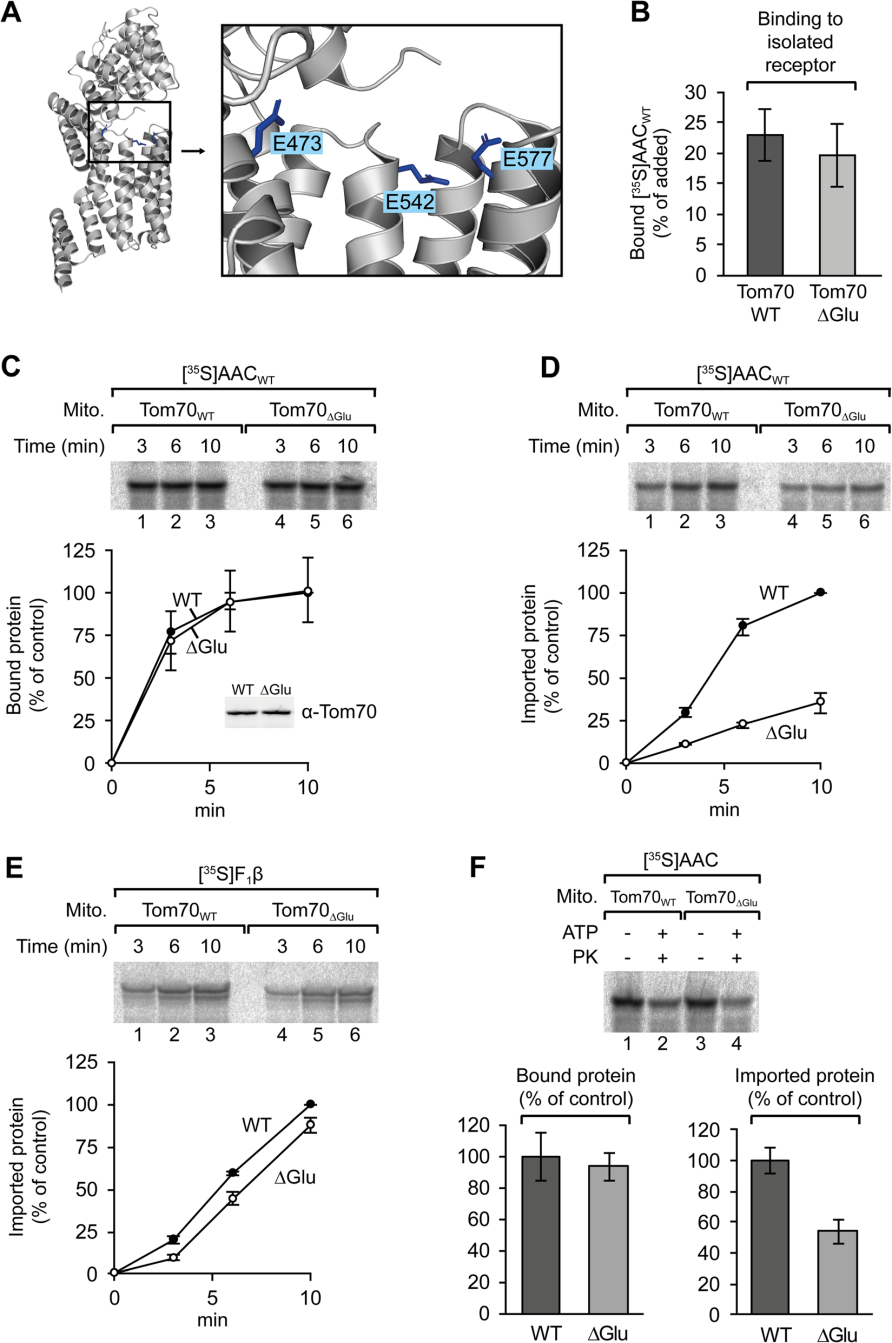
Role of Tom70 glutamate residues 473, 542, and 577 in binding and import of AAC. **a** Location of glutamate residues E473, E542, and E577 in Tom70. The structural data were taken from Wu and Sha [53], PDB 2GW1. **b** Binding of [^35^S]-labeled AAC to Ni-NTA-bound wild type Tom70 (Tom70_cd_) or Tom70_ΔGlu_ (E473Q/E542Q/E577Q), SD, *n* = 6. **c** Time course of binding of [^35^S]-labeled AAC to isolated yeast mitochondria containing either wild type Tom70 (Tom70_WT_) or Tom70_ΔGlu_, respectively. SD, *n* = 3. The relative amounts of mitochondrial Tom70 were assessed by immunoblotting (insert). **d** Import of AAC into isolated yeast mitochondria containing Tom70_WT_ or Tom70_ΔGlu_, respectively. The mitochondria were incubated with [^35^S]-labeled AAC in presence of ATP at 25 °C and subsequently treated with proteinase K. The amounts of imported AAC in mitochondria containing Tom70_WT_ within 10 min were set to 100%, SD, *n* = 3. **e** Mitochondrial import of F_1_β. The experiment followed the same procedure as in **d**. The amounts of imported F_1_β in mitochondria containing Tom70_WT_ within 10 min were set to 100%, SD, *n* = 3. **f** Binding of AAC to mitochondria and subsequent import (binding and chase). Isolated mitochondria containing wild type Tom70 or Tom70_ΔGlu_ were incubated with [^35^S]-labeled AAC in ATP-depleted reticulocyte lysate for 10 min at 25 °C, and reisolated, resuspended in buffer, and the suspensions were split into equal aliquots. The mitochondria were then again reisolated to determine the amounts of bound AAC (binding) or incubated in presence of 2 mM ATP and reisolated (chase), SD, *n* = 3

The C-terminal domain of yeast Tom70 contains three highly conserved glutamate residues, Glu473, Glu542, and Glu577 (Fig. 4a), and it was proposed that the receptor function of Tom70 may depend on these residues [53]. We exchanged these residues against glutamine and isolated the modified protein (Tom70_ΔGlu_) in parallel to the wild type protein (Tom70_cd_). Upon stepwise degradation with trypsin, the pattern of peptide fragments was similar for both proteins, suggesting that the basic folding state of the modified protein was retained (Additional file 3: Fig. S3). In tests for binding of radiolabeled AAC, the relative amounts of associated AAC were similar for wild type Tom70 and for the modified receptor lacking the glutamate residues (Fig. 4b). Correspondingly, binding of radiolabeled AAC to isolated mitochondria gave similar results with wild type mitochondria and with mitochondria carrying the modified receptor (Fig. 4c). However, a difference between the two receptor proteins was observed in import experiments (Fig. 4d). In this case, the mitochondria were incubated with the AAC in the presence of ATP and the mitochondria were subsequently treated with proteinase K. Comparing the relative amounts of protease-protected protein, we found that mitochondria containing the modified receptor Tom70_ΔGlu_, imported only about 40% radiolabeled AAC as compared to the mitochondria containing the wild type protein (Fig. 4d). Mitochondria of the same preparation showed only minor deficiencies in the import of the precursor of F_1_β (a presequence-targeted protein [59]) (Fig. 4e). The glutamate residues Glu473, Glu542, and Glu577 of Tom70 are obviously not part of a major binding site for the AAC. However, the glutamate residues are required for efficient transfer of the AAC from Tom70 to the import channel, possibly due to a function in structural rearrangements within Tom70 that are involved in the release of the bound AAC [57, 58].

The relevance of the glutamate residues in the release of the AAC was confirmed in experiments with consecutive steps of binding to the mitochondrial surface and subsequent translocation across the outer membrane (Fig. 4f). The results demonstrate that Tom70 has not only a defined affinity, but also a defined capability to release its substrates.

### Mitochondrial targeting by different AAC modules

We synthesized DHFR fusion proteins of the three AAC modules and compared the efficiencies of binding to isolated Tom70_cd_ (Additional file 4: Fig. S4A). In these assays, module III showed the highest affinity; for modules II and I, the values were significantly lower. Differences between the three modules were also observed in binding to isolated mitochondria (Additional file 4: Fig. S4B): A series of constructs lacking the positive charges in defined matrix loops was tested for binding to compare the contribution of each module in the context of the other two modules. The data show that the modules cooperate in binding, targeting is not dependent on a single unit. Comparing the binding of the AAC constructs to trypsin-pretreated and to untreated mitochondria, we found that also in the absence of Tom70, the three modules of the AAC are able to cooperate in binding to the mitochondria.

**Fig. 5:**
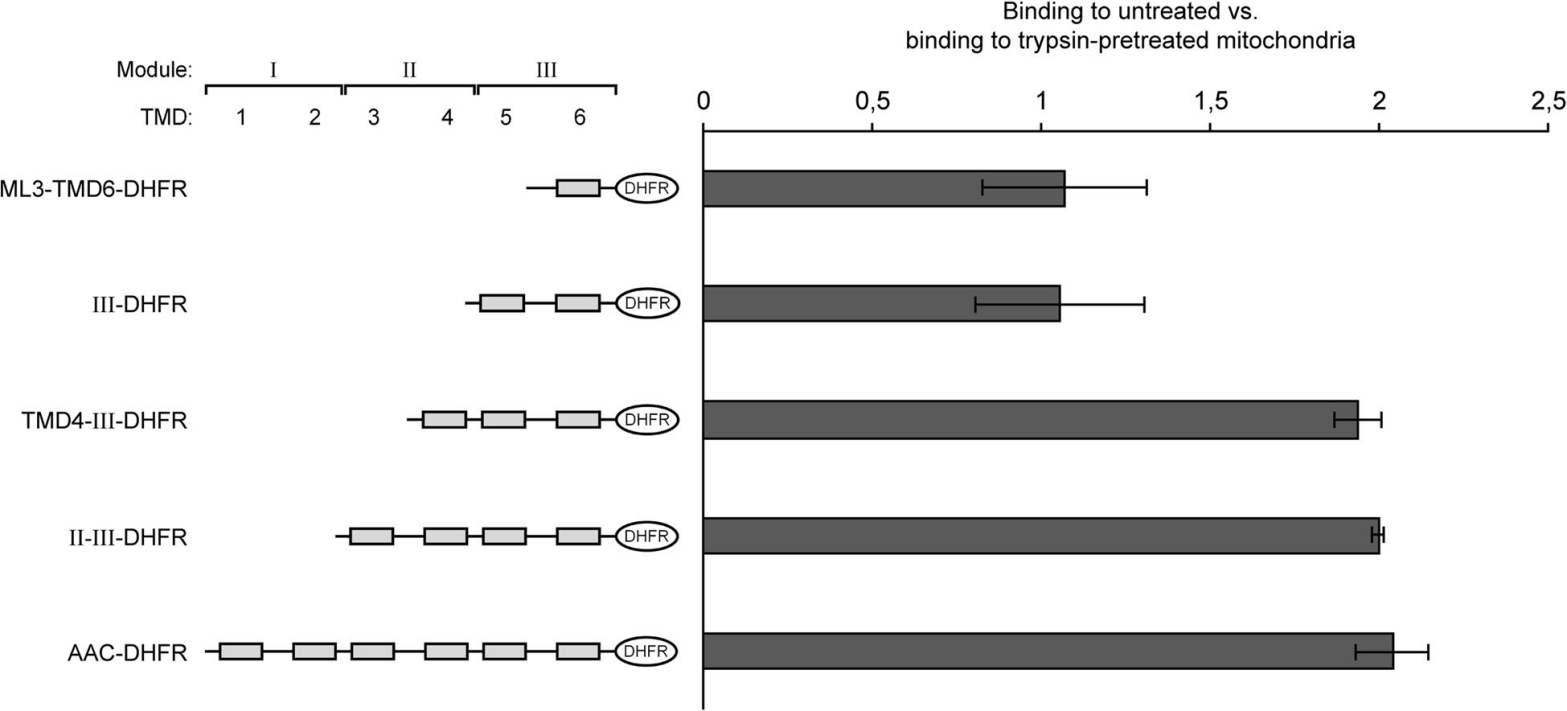
Binding of C-terminal segments of AAC to isolated mitochondria. [^35^S]-labeled segments of the AAC fused to DHFR were synthesized in reticulocyte lysate and incubated with isolated yeast mitochondria. The samples were ATP-depleted by incubation with apyrase. The mitochondria were reisolated, and the relative amounts of radiolabeled polypeptides were determined. The amounts of radiolabeled protein bound to untreated mitochondria were compared to the average amounts of the same protein bound to trypsin-pretreated mitochondria. SD, *n* = 3. TMD, transmembrane domain; ML3, matrix loop of module III

Eventually, we tested if the length of a polypeptide plays a role in the participation of the trypsin-sensitive receptor proteins in binding to the mitochondria (Fig. 5). Shortened fragments of the AAC were again fused to DHFR to facilitate the resolution in SDS-PAGE. Surprisingly, the smallest fragments, comprising only module III or only the bipartite targeting sequence, bound in similar amounts to the trypsin-treated and to the untreated mitochondria. In these assays, both constructs did not show any evidence of an interaction with Tom70. An involvement of trypsin-sensitive sites required at least three membrane-spanning helices (Fig. 5, constructs III-DHFR vs. TM4-III-DHFR).

### Bipartite mitochondrial targeting signals in Ant1p and in Ugo1

**Fig. 6:**
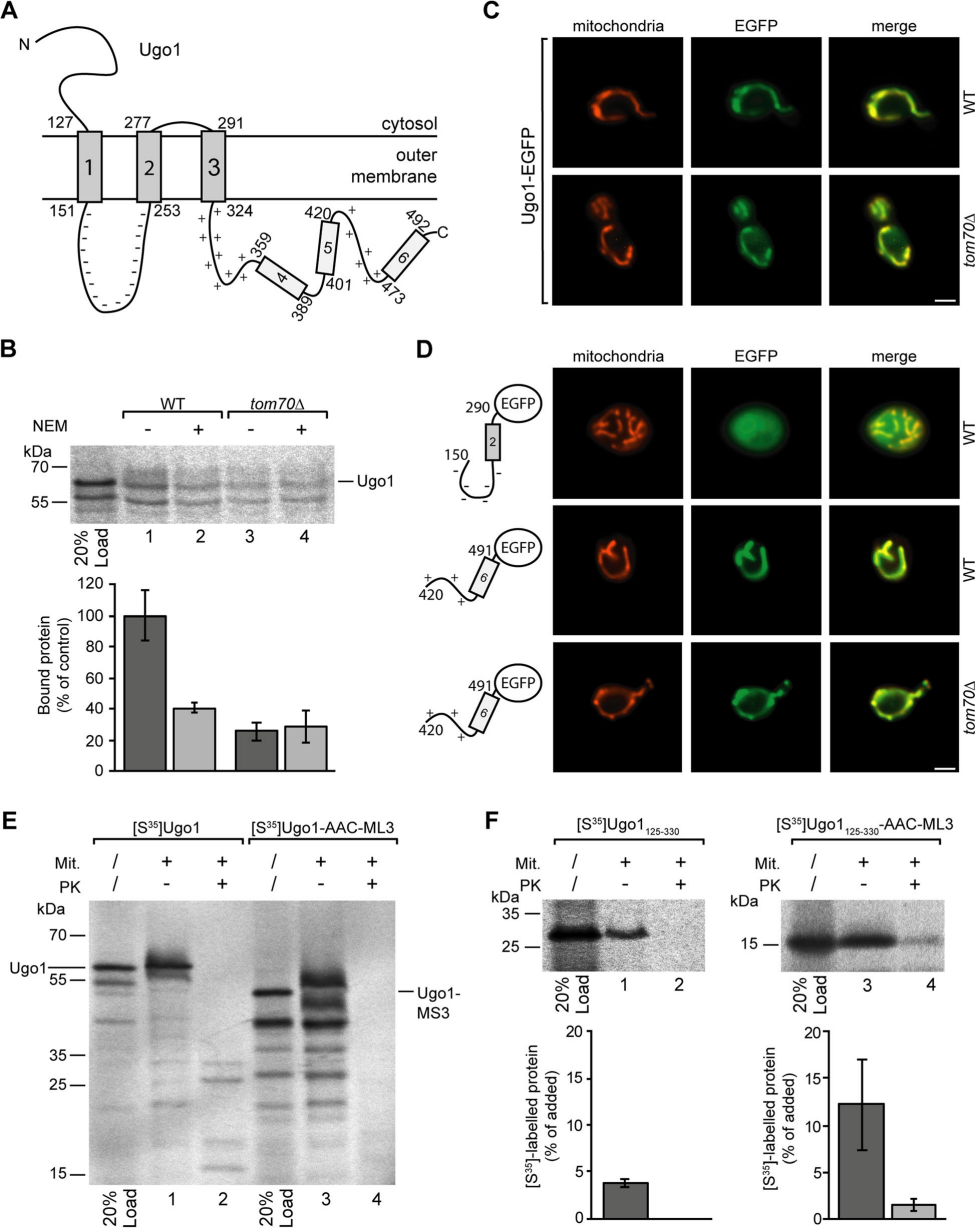
Mitochondrial targeting of Ugo1. **a** Topology of Ugo1 (as described by Hoppins et al., 2009 [60]). **b** Binding of [^35^S]-labeled Ugo1 to mitochondria isolated from yeast wild type cells (WT) or from a *tom70*Δ deletion strain. The mitochondria of samples 2 and 4 were pretreated with 2 mM NEM as described in the legend of Fig. 3. The average amount of Ugo1 bound to wild type mitochondria in the absence of NEM was set to 100% (control), SD, *n* = 4. **c** Fluorescence microscopy of EGFP-labeled Ugo1 expressed in yeast wild type cells (upper panel) and in cells of a *tom70*Δ deletion strain (lower panel). Mitochondria are labeled by MitoTracker Orange; bar 2 μm. **d** Images of yeast cells expressing different segments of Ugo1 labeled by a EGFP moiety expressed in wild type and in *tom70*Δ cells, respectively. **e** Incubation of isolated mitochondria with radiolabeled Ugo1 and a modified version of Ugo1 containing a substitution of residues 151–253 against the matrix loop of AAC module III (ML3, AAC residues 238–277). The mitochondria of samples 2 and 4 were subsequently treated with proteinase K (PK). Following SDS-PAGE, the radiolabeled polypeptides were visualized using the phosphorimager. **f** A [^35^S]-labeled fragment of Ugo1 (residues 125–330) and a modified version of this fragment (Ugo1125-330-AAC-ML3) containing the substitution of the negatively charged loop against the matrix loop of AAC module III were incubated with mitochondria as in **e**. The phosphorimager was used for quantification, and relative amounts of added protein are shown, SD, *n* ≥ 9

To test the results of our experiments with the AAC, we used two MCF proteins, Ant1p and Ugo1 (Fig. 6 and Additional file 5: Fig. S5), that are not targeted to the mitochondrial inner membrane but to peroxisomes or to the mitochondrial outer membrane, respectively.

The yeast adenine nucleotide transporter Ant1p is a protein of 36.4 kDa (UniProtKB - Q06497). Ant1p is recognized by the cytosolic protein Pex19p and transported to peroxisomes [36]. However, the primary structure reveals that Ant1p is a member of the MCF family. The three modules of Ant1p contain hydrophilic loops showing an excess of positively charged residues, suggesting that the essential requirements for mitochondrial targeting should be fulfilled. In fact, we found that in wild type yeast cells, an EFGP-Ant1p fusion protein co-localized with peroxisomes visualized with reporter protein mCherry-SKL [61]. However, the same protein showed a clear mitochondrial localization in the cells of a *pex19Δ* deletion strain (Additional file 5: Fig. S5A), confirming that fully intact mitochondrial targeting sequences are retained in the Ant1p structure, despite the peroxisomal localization.

In agreement with this conclusion, Ant1p bound efficiently to Tom70 and to isolated mitochondria (Additional file 5: Fig. S5B-D). However, only minor amounts of Ant1p were imported into a protease-protected location inside the mitochondria, indicating that structural elements are missing in Ant1p that are essential in subsequent steps of mitochondrial import (Additional file 5: Fig. S5E).

Ugo1, a protein of 502 residues (58 kDa; UniProtKB - Q03327), interacts with Tom70, but bypassing Tom40, it is subsequently inserted into the outer membrane by a Mim1-dependent mechanism [35, 60, 62, 63]. The N-terminal half of Ugo1 is embedded in the outer membrane by three α-helices, and the entire C-terminal part is localized in the intermembrane space [60] (Fig. 6a). Similar to the AAC, we found that the interactions of Ugo1 with Tom70 could be completely inhibited by pretreatment of the mitochondria with NEM (Fig. 6b); however, targeting was also possible independently of Tom70 (Fig. 6c).

As shown in Fig. 6d, the negatively charged loop (residues 151–253) of Ugo1 together with the adjacent transmembrane helix 2 did not bind to mitochondria, but a C-terminal segment (residues 420–491) comprising a positively charged sequence together with a hydrophobic segment mediated a distinct mitochondrial localization.

The negatively charged loop between helices 1 and 2 is essential in the integration of Ugo1 into the outer membrane (Fig. 6e, lane 4 vs. lane 2), but we also confirmed that a central fragment of Ugo1 (residues 125–330) containing the three membrane-spanning domains is not sufficient to mediate membrane integration (Fig. 6f, lane 2). However, when the negatively charged intermembrane space loop of this construct was exchanged against the positively charged matrix loop of AAC module III, the efficiency of mitochondrial targeting was increased about threefold, and a small but significant fraction of the construct was completely protected against the externally added proteinase K (Fig. 6f, lanes 3 and 4) and thus directed to the Tom40 import channel for import into the interior of the mitochondria. The membrane-embedded part of the outer membrane protein Ugo1 was redirected to the general import pathway of normal MCF proteins.

## Discussion

In this study, we investigated the mitochondrial targeting of the ADP/ATP carrier (AAC) and found that both the hydrophilic matrix loop and the C-terminal transmembrane helix of a module are necessary for efficient and specific targeting. The scheme of this bipartite targeting sequence is conserved in all members of the mitochondrial carrier family (MCF).

We found that the positive charges in the matrix loops are indispensable in mitochondrial targeting. This principle was confirmed in parallel experiments with the VMC1 (data not shown), an only distantly related virus-encoded MCF protein [64]. Within the AAC, the hydrophilic segment between helices 5 and 6 contains 40 residues and carries 4 negative and 8 positive charges. The N-terminal part of the segment contains a conserved motif, the carrier signature PX[D/E]XX[K/R] [40, 65]. We found that a shortened construct lacking this part fully retained its targeting function.

Tom70 was implicated in the recognition of the positively charged presequence of a mitochondrial protein [66] and similar internal structures [52]. Presequences of mitochondrial proteins are devoid of any negatively charged amino acid; they have a tendency to form amphipathic α-helices, and they are able to act as autonomous mitochondrial targeting signals [16]. None of these requirements is fulfilled by the matrix loops of the MCF proteins. We therefore conclude that the MCF matrix loops are elements of targeting signals with unique features and do not act in analogy to positively charged presequences. Correspondingly, the internal bipartite targeting signals of the MCF proteins also differ from the N-terminal bipartite targeting signals of other mitochondrial proteins such as yeast cytochrome c_1_ or NADH-cytochrome b_5_ reductase that contain a hydrophobic membrane-spanning domain connected to a classical amino-terminal presequence [67, 68].

Different from the AAC matrix loop, the adjacent transmembrane segment showed a significant affinity for the isolated import receptor Tom70 in vitro, and a fusion protein of AAC helix 6 and EGFP showed a partial co-localization with mitochondria of intact cells. However, the construct was mainly cytosolic and also some additional cellular structures were labeled. Hence, the transmembrane segment alone is not sufficient for specific mitochondrial targeting.

### The target of the targeting sequence

What is the target of the MCF targeting sequence? Prior to interactions with Tom70, newly synthesized preproteins are initially associated with cytosolic chaperone proteins, in particular with Hsp70 [69–72]. The N-terminal part of Tom70 contains a binding site for Hsp70, in yeast marked by the arginine of position 171 [55, 56], and we found that binding of AAC to mitochondrial Tom70 was completely blocked if the cysteine of position 141—in the vicinity of Arg171—was modified by reaction with NEM. Ser174, in close proximity, is an important phosphorylation site in the regulation of mitochondrial protein import [73]. The same region within the amino-terminal domain of Tom70, defined by residues C141, R171, and S174, is thus essential in binding of MCF proteins, relevant in binding of Hsp70, and modified by phosphorylation in the regulation of protein import. Yet, it has been unclear if the polypeptide chains of Hsp70-bound MCF proteins simultaneously also get into direct contact with Tom70.

The AAC can efficiently be crosslinked to Tom70 [29, 31, 74, 75]. However, the direct vicinity of both crosslinking partners is not necessarily due to specific interactions at defined binding sites. Considering that Hsp70 preferentially binds to hydrophobic segments [76, 77], it is conceivable that hydrophobic parts of the AAC associate with Hsp70 and bind to Tom70 only indirectly, leaving the hydrophilic segments of the AAC accessible for direct interactions with other interaction sites. Candidates for direct interactions are the three highly conserved glutamate residues Glu473, Glu542, and Glu577 of Tom70 [53]. However, we found that the efficiency of AAC binding to Tom70 was not significantly reduced after exchange of the glutamate residues against glutamine.

Comparing the affinities of different segments of the AAC, we encountered a puzzling contradiction: A modified AAC devoid of the positively charges in the three matrix loops showed no binding to mitochondria but a considerable binding to isolated Tom70. The isolated receptor is obviously lacking a decisive capability to recognize the positively charged matrix loops; this capability is dependent on additional structures that are not contained in the isolated receptor protein. Several lines of evidence indicate that the recognition of the positively charged matrix loop of the MCF targeting sequences is specifically due to interactions with Tom40: (1) In our experiments, even trypsin-pretreated mitochondria showed binding of wild type AAC, but no binding of the modified AAC_Δpos_ lacking the positive charges in the matrix loops, demonstrating that the essential and specific binding site is fully retained in the protease-treated mitochondria, a feature that is characteristic of the general import pore provided by Tom40. (2) Tom40 forms an ion channel with a clear preference for cations [78, 79] and is therefore an attractive candidate for the recognition of the positively charged matrix loops of the MCF proteins. (3) Tom40 was already shown in several independent studies to interact with internal parts of the AAC and of other MCF proteins [8, 29–31, 80].

Shiota et al. [80] used the method of chemical crosslinking to determine precisely the location of an AAC polypeptide chain in contact with Tom40. They found that the polypeptide chain was located in the vicinity of both hydrophobic patches and acidic residues inside the Tom40 channel. More recently, Araiso et al. [8] confirmed that translocating polypeptides pass both hydrophobic sites and acidic patches. These data support the conclusion that Tom40 should not only be able to recognize the positively charged matrix loops, but also the hydrophobic segments of the bipartite MCF targeting signals and to act as an autonomous selectivity filter.

Similar to MCF proteins, also presequence-targeted preproteins can be imported independently of mitochondrial outer membrane import receptors [23, 25, 26]. Considering that several independent studies identified binding sites for presequences in Tom40 [8, 80–84], it is tempting to assume that also in presequence-dependent protein import, Tom40 may form the major site of selectivity. Following this line, Tom40 would not only form a general import pore (GIP [85, 86]) but also a general selectivity filter, a GSF of mitochondrial protein import, and thereby determine which proteins are incorporated into the mitochondrial proteome. In this regard, it remains to be established if, or to which extent, this system is bypassed by some proteins of the mitochondrial outer membrane and the intermembrane space that follow separate import pathways [5, 17, 87].

### Transfer to Tom40

MCF proteins bind to Tom70 preferentially by their C-terminal module. Due to their lower affinities, the N-terminal modules have a higher mobility and screen the surrounding for subsequent interaction sites; hydrolysis of Hsp70-bound ATP then initiates the release from Tom70. We found that fragments of MCF proteins require at least three hydrophobic helices for productive cooperation of Tom70 with Tom40. Translocation through the Tom40 import channel is driven by the complex of Tim9 and Tim10 of the intermembrane space [88, 89]. This step is dependent on hydrophobic interactions with the transmembrane domains of the MCF proteins, the positively charged matrix loops seem not to play an important role [90, 91].

We found that also the C-terminal part of the outer membrane MCF protein Ugo1 can bind to Tom70. However, a positively charged loop is missing in the N-terminal sections of the protein, the two N-terminal helices of Ugo1 are connected by a negatively charged segment and interactions with the Tom40 import pore are blocked. In this case, the outer membrane protein Mim1 initiates an insertion into the mitochondrial outer membrane [62]. Interestingly, parts of several presequence-targeted preproteins initially bind to Tom70 to facilitate subsequent interactions of the presequences with Tom20 for efficient transfer to downstream components of the mitochondrial import machineries [92]. Tom70 appears to offer a docking site for different preproteins that subsequently proceed on different import pathways.

### The function of Tom70

Tom70 is an abundant protein of the mitochondrial outer membrane and was originally described as one of two import receptors, Tom70 and Tom20, each with a specificity for a distinct subset of preproteins [22]. But what is the function of Tom70 in mitochondrial protein targeting? The main contribution of Tom70 is assumed to be a facilitated binding of MCF proteins to mitochondria. In agreement with published data [23], we observed in most of our experiments that Tom70 mediated an about 2-fold increase in the amounts of AAC bound to isolated mitochondria. However, the specificity of mitochondrial targeting seems to be entirely independent of Tom70. Isolated Tom70 alone is not able to distinguish the authentic AAC from a derivative lacking the positively charged residues in the three matrix loops. This task is undertaken with high precision by mitochondria even in the absence of all external protease-accessible receptor sites. The biogenesis of mitochondria in plants is generally independent of any Tom70 homologs [93].

What then is the function of Tom70? Contrary to previous assumptions, Tom70 is not a specific receptor protein with a function restricted to a role in mitochondrial protein import. Primarily it acts as an abundant and versatile adaptor protein, suitable to facilitate the cooperation of mitochondria with different interaction partners. It was found in ER-mitochondria contact sites and implicated in sterol transport [94, 95] and in Ca^2+^ transfer [96]. Other data indicate a role of Tom70 in splicing of tRNA [97]. In mitochondrial protein import, Tom70 seems to act mainly as a co-chaperone, helping in the recruitment of chaperone-bound preproteins. In this regard, Tom70 shows impressive similarities to the protein Sec72 that binds Hsp70-associated proteins at the outer surface of the endoplasmic reticulum (ER). Sec72 exposes a tetratricopeptide repeat (TPR) domain analogous to the TPR domains contained in Tom70. Crystal structures revealed that within the Sec72 complexes, Hsp70 binds directly to the TPR domain [98].

## Conclusions

The targeting of newly synthesized MCF proteins is obviously a matter of consecutive steps: A first decision is already attained in binding to distinct chaperone proteins. If a newly synthesized protein binds to Pex19p, it can be targeted to peroxisomes as demonstrated by Ant1p. Hsp70-bound preproteins lacking a high affinity Pex19p interaction site can get into contact with mitochondrial Tom70 and bind at the site marked by cysteine C141. However, their subsequent fate is mainly determined by interactions with Tom40 that serves both as a general import pore and as a general selectivity filter: MCF proteins exposing a positively charged matrix loop, as well as preproteins containing a positively charged presequence, will be recognized by Tom40 and insert into the import pore. Tom70-bound proteins containing a negatively charged loop, such as Ugo1, may be expelled from the Tom40 import channel and insert into the lipid phase of the mitochondrial outer membrane, other proteins may be released and stay in the cytosol. Further studies on the selection and translocation of carrier proteins should be facilitated by the new data on the high-resolution structures of the import machinery that have recently become available, and by the identification of the bipartite MCF targeting sequence.

## Methods

### Yeast strains

The *Saccharomyces cerevisiae* strains used in this study are listed in Table 1. In order to create BY4742*tom70*∆/*tom71*∆, a DNA cassette encoding a natMX6 selection marker was amplified from the plasmid pAG25 [99] via PCR using a Phusion polymerase (Thermo Fisher Scientific) and introduced into the *tom70* gene of BY4742*tom71*∆ by homologous recombination. Plasmids, empty vector controls, and DNA cassettes were transformed into corresponding yeast strains via the lithium acetate method using carrier DNA [100]. Yeast cells were grown on YP medium (2% [w/v] peptone; 1% [w/v] yeast extract) supplied with either 2% [w/v] glucose (YPD; pH 6.5) or for induction of growth under respiratory conditions 3% [w/v] glycerol (YPG; pH 5.0) as a non-fermentable carbon source. For growth under selection pressure, yeast cells were grown on a selective complete medium containing an amino acid mixture lacking the amino acid of the selection marker (SD: 2% [w/v] glucose; 0.5% [w/v] (NH_4_)_2_SO_4_; 0.17% [w/v] yeast nitrogen base; 0.1% [w/v] amino acid mixture; pH 6.25). Cells were typically grown at 30 °C to an early exponential growth phase, whereas the optical density was determined with a photometer at a wavelength of 600 nm (OD_600_).

**Table 1:** *S. cerevisiae* strains used in this study

Yeast strains	Source	Acc. No.
BY4742 (WT) MATα *his*3Δ1 *leu*2Δ0 *lys*2Δ0 *ura*3Δ0	[101]	Y10000
BY4742 *tom70*::kanMX4	EUROSCARF	Y17244
BY4742 *pex19*::kanMX4	EUROSCARF	Y13762
K39 (CEN.PK 111-61A) MATα *ura*3–52 *leu*2–3.1/2 *his*3Δ1 *MAL*2-8c *MAL*3 *SUC*3 *GAL*	Prof. Heinisch, Osnabrück (D)	
K39 pUG35-AAC (*Neurospora crassa*)	This study	
K39 pUG36-AAC∆pos	This study	
K39 pUG35-AAC(221–313)	This study	
K39 pUG35-AAC∆pos(221–313)	This study	
K39 pUG36-AAC(238–313)	This study	
K39 pUG36-AAC(278–313)	This study	
K39 pUG36-AAC(238–277)	This study	
K39 pUG36-AAC(238–305)	This study	
K39 pUG35-AAC(221–277)	This study	
K39 pUG35-AAC(278–296 + 238–277)	This study	
K39 pUG36-AAC(238–277 + 221–237)	This study	
K39 pUG36-AAC(238–313)R290Q	This study	
K39 pUG36-AAC(238–300)	This study	
K39 pUG36-AAC(238–290)	This study	
K39 pUG36-AAC(246–313)	This study	
K39 pUG36-AAC(258–313)	This study	
K39 pUG36-AAC(33–97)	This study	
K39 pUG36-AAC(138–201)	This study	
K39 pUG36-DIC(227–298) (*Saccharomyces cerevisiae*)	This study	
BY4742 *tom70*::kanMX4 pUG35-AAC	This study	
BY4742 *tom70*::kanMX4 pUG36-AAC(238–305)	This study	
BY4742 *tom70*::kanMX4 pPC97-Tom70WT	This study	
BY4742 *tom70*::kanMX4 pPC97-Tom70C141A	This study	
BY4742 *tom70*::kanMX4 pPC97-Tom70C439A	This study	
BY4742 *tom70*::kanMX4 pPC97-Tom70C450A	This study	
BY4742 *tom70*::kanMX4 pPC97-Tom70E473,542,577Q	This study	
BY4742 pUG35-Ugo1 (*Saccharomyces cerevisiae*)	This study	
BY4742 *tom70*::kanMX4 pUG35-Ugo1	This study	
BY4742 pUG35-Ugo1(150–290)	This study	
BY4742 *tom70*::kanMX4 pUG35-Ugo1(420–491)	This study	
BY4742 (*tom70*∆/*tom71*∆) *tom70*::kanMX4 *tom71*::natMX6	This study	
BY4742 *tom70*∆/*tom71*∆ pUG35-AAC (*Neurospora crassa*)	This study	
BY4742 *tom70*∆/*tom71*∆ pUG36-AAC(238–305)	This study	

### Plasmid construction

Plasmids and oligonucleotides used for recombinant DNA construction are listed in Table 2. For general plasmid construction, DNA fragments were amplified by standard PCR using the appropriate primer pair and a Phusion polymerase (Thermo Fisher Scientific). After restriction enzyme digestion, inserts were ligated into vectors using the T4 DNA ligase (Thermo Fisher Scientific) following the manufacturer’s recommendations.

**Table 2:** Plasmids used in this study

Plasmids	Source
pUG35	Güldener & Hegemann, Heinrich Heine University Düsseldorf, (D)
pUG36	Güldener & Hegemann, Heinrich Heine University Düsseldorf, (D)
pAG25-natMX6	[99]
pYES2	Invitrogen, Karlsruhe (D)
pET10N	[102]
pPC97	[103]
pGEM-4Z	Promega
pGEM-11Zf(+)	Promega
pUG35-AAC (*Neurospora crassa*)	This study
pUG36-AAC∆pos	This study
pUG35-AAC(221–313)	This study
pUG35-AAC∆pos(221–313)	This study
pUG36-AAC(238–313)	This study
pUG36-AAC(278–313)	This study
pUG36-AAC(238–277)	This study
pUG36-AAC(238–305)	This study
pUG35-AAC(221–277)	This study
pUG35-AAC(278–296 + 238–277)	This study
pUG36-AAC(238–277 + 221–237)	This study
pUG36-AAC(238–313)R290Q	This study
pUG36-AAC(238–300)	This study
pUG36-AAC(238–290)	This study
pUG36-AAC(246–313)	This study
pUG36-AAC(258–313)	This study
pUG36-AAC(33–97)	This study
pUG36-AAC(138–201)	This study
pUG36-DIC(227–298) (*Saccharomyces cerevisiae*)	This study
pPC97-Tom70WT	This study
pPC97-Tom70C141A	This study
pPC97-Tom70C439A	This study
pPC97-Tom70C450A	This study
pPC97-Tom70E473,542,577Q	This study
pET10N-Tom70(38–617)	This study
pET10N-Tom70(38–617)C141A	This study
pET10N-Tom70(38–617)C439A	This study
pET10N-Tom70(38–617)C450A	This study
pET10N-Tom70(38–617)E473,542,577Q	This study
pUG36-Ant1 (*Saccharomyces cerevisiae*)	[36]
pUG35-Ugo1 (*Saccharomyces cerevisiae*)	This study
pUG35-Ugo1(150–290)	This study
pUG35-Ugo1(420–491)	This study
pDS5/2-Su9-DHFR (ATP synthase subunit 9 1–69, *Neurospora crassa*; DHFR, mouse)	[104]
pGEM-4Z-F1β (*Neurospora crassa*)	[59]
pGEM-11Zf(+)-AAC (*Neurospora crassa*)	This study
pGEM-11Zf(+)-AAC-NseI-BamHI	This study
pGEM-11Zf(+)-AAC∆pos	This study
pGEM-11Zf(+)-AAC-I∆pos-IIwt-IIIwt	This study
pGEM-11Zf(+)-AAC-Iwt-IIwt-III∆pos	This study
pGEM-11Zf(+)-AAC-I∆pos-II∆pos -IIIwt	This study
pGEM-11Zf(+)-AAC-Iwt-II∆pos -III∆pos	This study
pYES2-DHFR	This study
pYES2-AAC2 (*Saccharomyces cerevisiae*)	This study
pYES2-AAC-DHFR	This study
pYES2-AAC(117–313)-DHFR	This study
pYES2-AAC(183–313)-DHFR	This study
pYES2-AAC(216–305)-DHFR	This study
pYES2-AAC(238–313)-DHFR	This study
pYES2-AAC(278–313)-DHFR	This study
pYES2-AAC(238–277)-DHFR	This study
pYES2-AAC(10–100)-DHFR	This study
pYES2-AAC(117–206)-DHFR	This study
pYES2-Ant1	This study
pYES2-Ugo1 (*Saccharomyces cerevisiae*)	This study
pYES2-Ugo1-AAC-ML3	This study
pYES2-Ugo1(125–330)	This study
pYES2-Ugo1(125–330)-AAC-ML3	This study
mCherry-SKL	[61]

For creation of pGEM-11Zf(+)-AAC∆pos, a synthesized gene of *Neurospora crassa* AAC in which all positively charged amino acids in its three matrix loops were exchanged against glycines (all K,R→G in regions aa35-aa78, aa140-aa182, and aa240-aa277) was introduced into vector pGEM-11Zf(+) between HindIII_fwd and XhoI_rev bringing it under control of a SP6 promotor. The gene contained restriction sites for NseI (nt335-nt341 in respect to coding reading frame) and BamHI (nt703–709). For the creation of hybrid constructs (pGEM-11Zf(+)-AAC-I∆pos-IIwt-IIIwt, pGEM-11Zf(+)-AAC-Iwt-IIwt-III∆pos, pGEM-11Zf(+)-AAC-I∆pos-II∆pos-IIIwt, pGEM-11Zf(+)-AAC-Iwt-II∆pos-III∆pos), additional restriction sites were introduced into pGEM-11ZF(+)-AAC (NseI: nt335-nt341; BamHI: nt703–709) allowing exchange of single modules between constructs.

For creation of TMD exchange constructs (pUG36-AAC (238–277 + 221–237); pUG35-AAC (278–296 + 238–277)), an NheI restriction site was introduced either before TMD5 and after ML3, or after TMD6 and before ML3.

### Fluorescence microscopy of EGFP-labeled proteins in yeast

The desired DNA fragments were inserted into the vector pUG35 or pUG36 (Dr. Güldemann and Dr. Hegemann, University of Düsseldorf, Germany) for constitutive expression of a hybrid protein containing an enhanced green fluorescent protein yEGFP3 [39] from a MET25 promotor. Transformed yeast cells were grown in SD medium (2% [w/v] glucose; 0.1% [w/v] yeast extract; 0.5% [w/v] (NH_4_)_2_SO_4_; 0.17% [w/v] yeast extract; 0.1% [w/v] amino acid mixture) until an early exponential phase and washed twice with PBS (137 mM NaCl; 2.7 mM KCl; 10 mM Na_2_HPO_4_; 1.8 mM KH_2_PO_4_; pH 7.4). For fluorescence microscopy, an Axioplan 2 imaging microscope (Zeiss) with an alpha Plan Fluar × 100/1.45 objective under oil immersion was used. Mitochondria were labeled with MitoTracker Orange CMTMRos (Thermo Scientific, M7510) for 40–60 min at 30 °C following the manufacturer’s recommendations and visualized using a TRITC fluorescence filter at 25 °C. EGFP fluorescence was visualized using a GFP filter. Images were obtained with an AxioCam MRm camera (Zeiss) using the software AxioVision 4.6.3. and Zen 2.5 lite.

### Isolation of mitochondria from *Saccharomyces cerevisiae*

Mitochondria were isolated from the yeast strain BY4742 [101] (WT) or related strains, essentially following the procedures published by [105, 106].

#### Growth of the cells

The starter cultures contained glucose as a carbon source, and the main cultures contained glycerol (YPG medium: 1% [w/v] yeast extract, 2% [w/v] peptone, 3% [w/v] glycerol; pH 5.0). Media were autoclaved at 121 °C for 20 min. The main cultures had a volume of 2000 ml and were incubated in 5000-ml flasks at 30 °C under vigorous shaking (140 rpm) for 24 h to achieve an OD_600_ of 1–2. The cells were harvested by centrifugation for 5 min at 3000×*g* at room temperature. Excess medium was removed by resuspension of the cells in distilled water and subsequent centrifugation. Starting with a culture of a total volume of 24 l, about 20–60 g yeast cells were obtained.

#### Formation of spheroplasts

The cell pellet was resuspended in 1 ml Tris/DTT buffer (100 mM Tris-H_2_SO_4_ pH 9.4; 10 mM dithiothreitol) per 0.5 g wet weight of cells. The cell suspension was incubated for 30 min at 30 °C under gentle shaking and the cells were pelleted by centrifugation for 5 min at 3000×*g* at 4 °C. The cells were resuspended in 1.2 M sorbitol, reisolated by centrifugation, and then suspended in 1 ml 1.2 M sorbitol, 20 mM KH_2_PO_4_, and pH 7.4 per 0.15 g wet weight of cells. The suspension was supplemented with 1.5–2 mg zymolyase (20T, from *Arthrobacter luteus*; Amsbio, 120491-1) per gram wet weight of cells and incubated for 60 min at 30 °C under gentle shaking. The conversion of the cells into spheroplasts was monitored by observing the decrease of optical density of the suspension at 600 nm before and after zymolyase treatment. The spheroplasts were pelleted by centrifugation for 5 min at 3000×*g* at 4 °C. To remove the zymolyase, the spheroplasts were washed twice by resuspension in 1.2 M sorbitol and centrifugation.

#### Lysis of spheroplasts and isolation of mitochondria

The spheroplasts were suspended in 1 ml cold homogenization buffer (0.6 M sorbitol; 10 mM Tris-HCl; 1 mM EDTA; 0.5% [w/v] BSA [fatty acid-free; Sigma, 9003 T]; pH adjusted after solubilization of the BSA to 7.4) per 0.15 g wet weight of cells. PMSF (phenylmethylsulfonyl fluoride) was added from a 200 mM stock solution (in ethanol) to a final concentration of 1 mM. The spheroplasts were lysed by 10–20 strokes of a tightly fitting pestle in a dounce homogenizer cooled on ice. The homogenate was centrifuged twice for 5 min at 4 °C, first at 2000×*g* and then at 3000×*g*. The pelleted cell debris was discarded. The mitochondria were isolated from the supernatant by centrifugation for 10 min at 15,000×*g* at 4 °C. To improve the purity, the mitochondria were resuspended in SEM buffer (250 mM sucrose; 1 mM EDTA; 10 mM MOPS/KOH; pH 7.2), 1 mM PMSF was added from the 200 mM stock solution, and the suspension was centrifuged for 5 min at 3000×*g* at 4 °C. The pellet was discarded, and the mitochondria were isolated from the supernatant by centrifugation for 10 min at 15,000×*g* at 4 °C. The mitochondria were resuspended in 10 μl SEM per 1000 ml original yeast culture and the protein concentration was adjusted to 10 mg/ml based on a Bradford protein assay (Pierce Coomassie Plus (Bradford) Protein-Assay, Thermo Scientific, 23238) using bovine serum albumin as a standard (Pierce Bovine Serum Albumin Standard, 2 mg/ml, Thermo Scientific, 23210). The suspension of mitochondria was eventually frozen in aliquots in liquid nitrogen and stored at − 70 °C. The yield of the procedure was usually 0.2–0.5 mg mitochondrial protein per gram cells (wet weight).

### Protein import into isolated yeast mitochondria

Radiolabeled proteins were synthesized by coupled transcription and translation in commercially available reticulocyte lysate (TNT Coupled Reticulocyte Lysate System, Promega, L4600) in the presence of [^35^S]-methionine (added from a stock solution of 10 μCi/μl, Hartmann Analytic, SCM01/37) following the instructions of the manufacturer. The reaction was carried out for 120 min at 30 °C. Reticulocyte lysate containing the radiolabeled precursor protein was clarified by centrifugation (1 h; 20,000×*g*; 4 °C).

Protein import into mitochondria essentially followed the procedures as described in [106]. Reticulocyte lysates containing radiolabeled proteins were incubated with isolated yeast mitochondria in volumes of 50 μl, containing 30 μg mitochondrial protein, 1–5 μl reticulocyte lysate, and import buffer (10 mM MOPS; 250 mM sucrose; 80 mM KCl; 5 mM MgCl_2_; 2 mM KH_2_PO_4_; 3% [w/v] essentially fatty acid-free BSA; pH 7.2). To achieve efficient import of proteins into isolated mitochondria, in some experiments ATP and NADH were added to the samples to a final concentration of 2 mM each.

The samples were usually incubated for 10 min at 25 °C and subsequently cooled on ice. Mitochondria were recollected by centrifugation (10 min; 20,000×*g*; 4 °C), and the supernatants were removed. Residual reticulocyte lysate was removed by resuspending the mitochondria in 100 μl SEM buffer (250 mM sucrose; 1 mM EDTA; 10 mM MOPS-KOH; pH 7.2) and additional reisolation by centrifugation. The pellet was dissolved in sample buffer (50 mM Tris; 1% [w/v] SDS; 5% [v/v] 2-mercaptoethanol; 0.01% [w/v] bromophenol blue; 10% [w/v] glycerol). Samples were denatured for 5 min at 95 °C, and the proteins were separated by SDS-PAGE following standard procedures. The gels were dried and radiolabeled proteins were detected with image plates and read out by a Bas1800 II phosphorimager (Fujifilm Europe GmbH). Quantification of signals was performed using the software Aida Image Analyzer V.4.19 (Raytest). An internal standard sample (“load”) was included in each experiment for comparison. Replicates used for quantification were independent binding and import assays of incubation of isolated yeast mitochondria (wild type and mutant mitochondria) with radiolabeled precursor proteins. Individual data from independent experiments with *n* < 6 are listed in Additional file 6: Table S1.

Depending on the particular experiment, the assay comprised additional steps:

In some experiments, the mitochondria were preincubated with 30 μg trypsin (Sigma-Aldrich, T1426) per mg protein on ice for 10 min. Proteolysis was stopped by addition of 40 μg soybean trypsin inhibitor (Sigma-Aldrich, T9003) per 1 μg trypsin.

To determine the amounts of protease-protected proteins in the import assays, the samples containing mitochondria were cooled to 0 °C and incubated with 75 μg/ml proteinase K on ice for 10 min. In the assays with Ugo1, 100 μg/ml proteinase K were used. Proteolysis was stopped by addition of 4 mM PMSF (phenylmethylsulfonyl fluoride, final concentration) and incubation on ice for 5 min.

For opening of the mitochondrial outer membrane after in vitro protein import of radiolabeled proteins, mitochondria were collected by centrifugation (10 min; 20,000×*g*; 4 °C) and washed with 100 μl SEM buffer. The mitochondria were then resuspended in SEM or EM buffer (1 mM EDTA; 10 mM MOPS-KOH; pH 7.2) and incubated for 20 min at 25 °C. To determine the amount of imported protein, samples were treated with 75 μg/ml proteinase K on ice for 10 min. The reaction was stopped by addition of 4 mM PMSF (final concentration) and an incubation on ice for 5 min. The mitochondria were reisolated, resuspended in 100 μl SEM buffer, and again reisolated. The samples were resuspended in sample buffer and denatured for 5 min at 95 °C, and the proteins were separated by SDS-PAGE.

To analyze the binding of [^35^S]-labeled proteins to the outer surface of isolated mitochondria, both the reticulocyte lysate and the mitochondria were preincubated in parallel with apyrase (NEB, M0398L) at a final concentration of 25 U/ml for 20 min at 25 °C. The procedure followed the protocol of [31]. The amounts of aggregated proteins were determined by including samples omitting the addition of mitochondria, and the corresponding values were subtracted in calculating of the mitochondria-associated amounts of protein.

The mitochondrial membrane potential was dissipated by addition of 2 μM (final concentration) valinomycin, added from a 200 μM stock solution (in ethanol).

### Preparation of yeast total cell extracts

The extraction of proteins from yeast cells was performed by the method of Kushnirov [107]. Cells were incubated overnight in YPD (1% [w/v] yeast extract; 2% [w/v] peptone; 2% [w/v] glucose) or SD medium (2% [w/v] glucose; 0.1% [w/v] yeast extract; 0.5% [w/v] (NH_4_)_2_SO_4_, 0.17% [w/v] yeast extract; 0.1% [w/v] amino acid mixture) to OD_600_ between 2.5 and 10, and 1 ml of cell suspension was harvested by centrifugation (1 min; 2500×*g*; RT). Cells were washed with water and incubated in 100 μl 0.1 M NaOH for 10 min at RT. For complete lysis, 25 μl of 5× SDS sample buffer were added and cells were incubated for 5 min at 95 °C. After centrifugation (1 min; 20,000×*g*; RT), typically 10 μl of supernatant was analyzed by SDS-PAGE. Alternatively, the TCA method was used to extract yeast proteins. In this case, 300 μl of cell suspension with OD_600_ = 2 was resuspended in 300 μl of 50 mM KP_i_ (pH 7.4) followed by addition of 100 μl of 50% TCA. After incubation at for 30 min at − 70 °C, the precipitate was collected by centrifugation (10 min; 20,000×*g*; 4 °C) and washed twice with − 20 °C cold 80% acetone solution. The precipitate was dried and resuspended in 80 μl 1% [w/v] SDS/0.1 M NaOH. Prior to loading on a polyacrylamide gel, 20 μl of 5× SDS sample buffer (5% [w/v] SDS; 50% [v/v] glycerol; 0.05% [w/v] bromophenol blue; 25% [v/v] 2-mercaptoethanol; 250 mM Tris/HCl, pH 6.8) was added to the mixture, and samples were incubated for 5 min and 95 °C whereas typically 10 μl of a sample was analyzed for SDS-PAGE.

### Affinity purification of proteins

For purification of Tom70 variants, the N-terminally His-tagged cytosolic domain of the receptor, His-Tom70_38–617_, was expressed in *E. coli* BL21(DE3) by induction with 0.5 mM IPTG. Cells were resuspended in extraction buffer (20 mM Tris/HCl; 150 mM NaCl; 10 mM imidazole; 2 mM PMSF; protein inhibitors; pH 7.4), and the protein was isolated from cell extract by affinity purification with HisTrap High-Performance columns (Sigma-Aldrich) and an ÄKTA Start system (GE Healthcare) using standard wash buffer (20 mM Tris/HCl; 150 mM NaCl; 40 mM imidazole pH 7.4). Bound proteins were eluted with an imidazole gradient (40–500 mM imidazole). Protein containing fractions were pooled and further purified via size exclusion chromatography using an ÄKTA purifier system (GE Healthcare) with a Superose 12 10/300 GL column (GE Healthcare) and standard buffer (20 mM Tris/HCl; 150 mM NaCl; pH 7.9). The purity of isolated proteins was confirmed by SDS-PAGE and Coomassie staining.

Purification of small amounts of proteins for in vitro binding assays was performed with Ni^2+^-NTA beads (Protino, Macherey-Nagel, 745400) via the batch method. Briefly, extracts generated from small aliquot of *E. coli* BL21(DE3) expressing the His-tagged target protein were mixed with Ni^2+^-NTA beads and incubated for 1 h at 4 °C. After washing the beads five times with standard wash buffer (20 mM Tris/HCl; 150 mM NaCl; 40 mM imidazole), the protein concentration was determined and the protein was kept on ice until further use.

### In vitro binding assay

To investigate binding of different AAC constructs to isolated Tom70, we used an in vitro binding assay modified according to Brix et al. [24]. Purified Tom70 bound to Ni^2+^-NTA beads was washed three times with assay buffer (10 mM MOPS/KOH; 20 mM imidazole; 150 mM KCl; 1% [w/v] BSA; pH 7.2) prior to use. A reaction mixture containing 50 pmol of purified Tom70 per 100 μl volume was added to Mobicol columns (Mobitech, M1002), and the reaction was started by addition of typically 7% [v/v] reticulocyte lysate containing [^35^S]-methionine-labeled protein synthesized in reticulocyte lysate (Promega). After incubation for 40 min at 30 °C while shaking, the column bed was washed three times with assay buffer lacking BSA and the protein was eluted with elution buffer (20 mM Tris/HCl; 1 M imidazole; 500 mM NaCl; pH 7.9). Eluted protein was precipitated with TCA and analyzed by SDS-PAGE. For quantification, the software Aida Image Analyzer V.4.19 (Raytest) was used. An internal standard sample (“load”) was included in each experiment for comparison. Independent binding assays were carried out with separate samples in individual columns. Unspecific binding was assessed by incubation of radiolabeled proteins with Ni^2+^-NTA beads that had been incubated with *E. coli* lysate prior to the assay. Individual values from independent experiments with *n* < 6 are listed in Additional file 6: Table S1.

### NEM treatment

For pretreatment with NEM, isolated mitochondria were diluted in SEM buffer to a protein concentration of 1 mg/ml. NEM (100 mM in EtOH) was added to a final concentration of 2 mM. As a control, mitochondria treated with the same volume of EtOH were analyzed in parallel. The samples were incubated for 20 min at 25 °C and recollected by centrifugation (10 min; 20,000×*g*; 4 °C). Recollected mitochondria were washed with SEM and resuspended in appropriate buffer according to the experiment as described above.

For in vitro binding assays, proteins bound to Ni^2+^-NTA beads were pretreated with 2 mM NEM on ice for 10 min and washed three times with binding buffer as described prior to addition of [^35^S]-methionine-labeled protein.

### Polyacrylamide gel electrophoresis

Protein samples were denatured in SDS sample buffer (50 mM Tris; 1% [w/v] SDS; 5% [v/v] 2-mercaptoethanol; 0.01% [w/v] bromophenol blue; 10% [w/v] glycerol) and denatured for 5 min at 95 °C prior to loading onto polyacrylamide gels (12.5% [w/v] acrylamide; 0.33% bis-acrylamide [w/v]; 350 mM Bis-Tris; pH 6.5). Electrophoresis was performed in the presence of either MOPS buffer (50 mM MOPS; 50 mM Tris; 1 mM EDTA; 0.1% [w/v] SDS; 5 mM Na_2_SO_3_; pH 7.0) for proteins with a mass larger than 20 kDa or MES buffer (50 mM MES; 50 mM Tris; 1 mM EDTA; 0.1% [w/v] SDS; 5 mM Na_2_SO_3_; pH 7.0) for proteins with a mass smaller than 20 kDa for 10 min at 100 V and subsequently for approximately 1 h at 150 V. After drying the gels, protein signals were detected with image plates, read out by a Bas1800 II phosphorimager (Fujifilm Europe GmbH), and analyzed with Aida Image Analyzer V.4.19. Alternatively, after gel electrophoresis, proteins were visualized by staining with colloidal Coomassie (5% [w/v] aluminum sulfate 14–18 hydrate; 8% [v/v] phosphoric acid; 10% [v/v] EtOH; 0.02% [w/v] SERVA Blue G) for 1 h to overnight and de-staining with 2% [v/v] phosphoric acid in 10% [v/v] EtOH.

## Supplementary information

**Additional file 1:** Fig. S1 Data on the intracellular localization of different EGFP-labeled segments of AAC as determined by fluorescence microscopy.

**Additional file 2: **Fig. S2 [^35^S]-labeled proteins in correspondence to Fig. 2f and in vitro binding and protein import of ML3-TM6-DHFR in WT and *tom70∆* mitochondria.

**Additional file 3:** Fig. S3 Coomassie-stained SDS-PAGE of proteolytic fragments of isolated Tom70 (residues 38–617) obtained by incubation with trypsin.

**Additional file 4:** Fig. S4 Data on the binding of AAC modules I, II and III to isolated Tom70 and to isolated mitochondria.

**Additional file 5:** Fig. S5 Data on the targeting of the peroxisomal protein Ant1p.

**Additional file 6: **Table S1 Individual values for experiments with *n* < 6.

## Data Availability

All data generated or analyzed during this study are included in this published article and its supplementary data (Additional files 1, 2, 3, 4, 5, and 6).
