# Supplementary material for: The selectivity filter of the mitochondrial protein import machinery
Source: BMC Biol. 2020 Oct 29;18:156. doi: 10.1186/s12915-020-00888-z (PMC7596997; doi:10.1186/s12915-020-00888-z)
Supplement: Supplementary file 1 — Additional file 1: Fig. S1 Data on the intracellular localization of different EGFP-labeled segments of AAC as determined by fluorescence microscopy. [file 12915_2020_888_MOESM1_ESM.pdf]

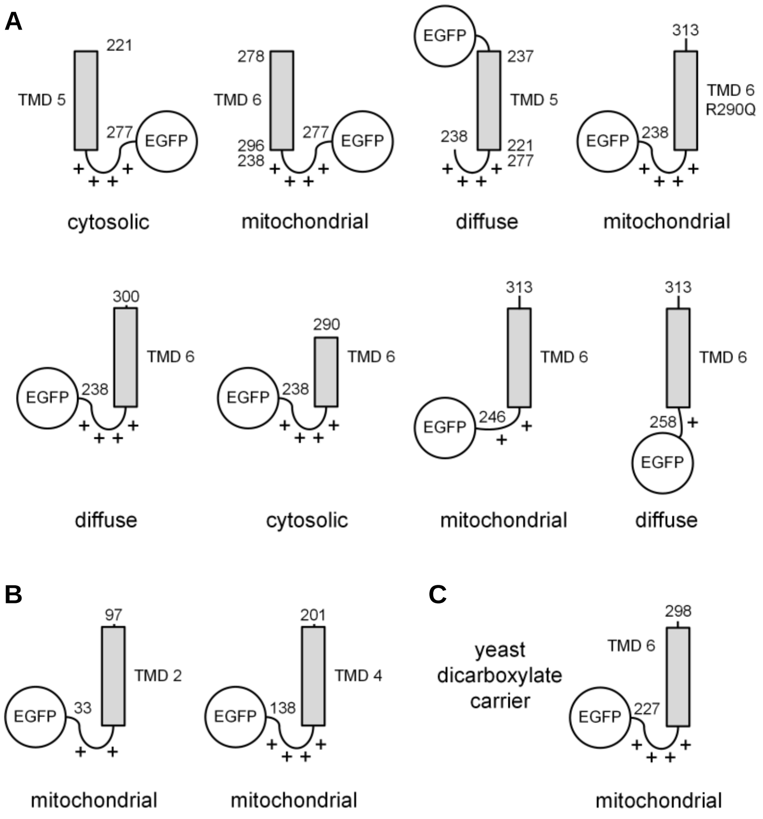

Figure S1. Intracellular localization of EGFP-labeled segments of AAC as determined by fluorescence microscopy.

A, Localization of EGFP-labeled segments of AAC module III and modified derivatives. The constructs were expressed in yeast and their localization was determined as described in legend to Figure 1. Indicated are the numbers of the first and the last residue of each polypeptide chain (with respect to the AAC primary structure). Constructs labeled “diffuse” showed mainly cytosolic localization with only partial labeling of mitochondria. **B**, Intracellular distribution of EGFP-labeled segments of AAC modules II or III, respectively. **C**, Localization of a segment of yeast dicarboxylate carrier (DIC1, UniProtKB – Q06143) module III.

Fig. S1
