# Supplementary material for: The selectivity filter of the mitochondrial protein import machinery
Source: BMC Biol. 2020 Oct 29;18:156. doi: 10.1186/s12915-020-00888-z (PMC7596997; doi:10.1186/s12915-020-00888-z)
Supplement: Supplementary file 2 — Additional file 2: Fig. S2 [35S]-labeled proteins in correspondence to Fig. 2f and in vitro binding and protein import of ML3-TM6-DHFR in WT and tom70∆ mitochondria. [file 12915_2020_888_MOESM2_ESM.pdf]

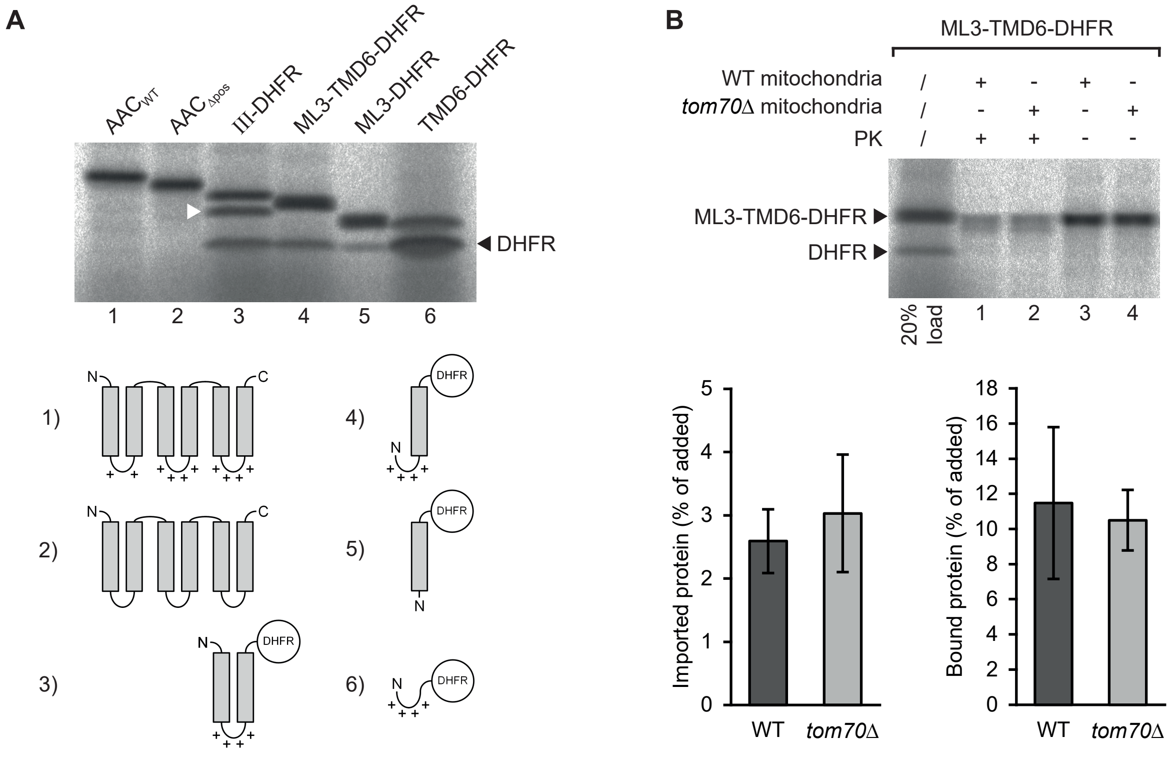

Figure S2. A, Synthesis of proteins in reticulocyte lysate in the presence of ^{35}S-methionine. AAC_{WT} ($AAC_{1\text{-}313}$; 1), AACΔpos ($AAC_{1\text{-}313}$, positively charged amino acids of the three matrix loops exchanged to glycine; 2) III-DHFR ($AAC_{221\text{-}313}$ fused to mouse dihydrofolate reductase (DHFR), the white arrow indicates translation product of a second reading frame of the plasmid; 3), ML3-TMD6-DHFR ($AAC_{238\text{-}313}$ fused to DHFR; 4), ML3-DHFR ($AAC_{238\text{-}277}$ fused to DHFR; 5), TMD6-DHFR ($AAC_{278\text{-}313}$ fused to DHFR; 6). **B**, Import of ML3-TMD6-DHFR into isolated mitochondria. Isolated wildtype mitochondria or mitochondria isolated from a yeast strain containing a deletion of the gene encoding Tom70 (*tom70Δ*) were incubated with [^{35}S]-labeled ML3-TMD6-DHFR in reticulocyte lysate for 10 min at 25 °C, reisolated and resuspended in SEM buffer. For assessment of the amount of imported protein, mitochondria were incubated in the presence of 75 µg/ml proteinase K on ice for 10 min and the reaction was subsequently stopped by addition of 4 mM PMSF on ice for 5 min. Mitochondria were reisolated, washed with SEM buffer and subjected to SDS-PAGE to determine the amounts of bound and imported protein, SD, n=3.

Fig. S2
