# Supplementary material for: The selectivity filter of the mitochondrial protein import machinery
Source: BMC Biol. 2020 Oct 29;18:156. doi: 10.1186/s12915-020-00888-z (PMC7596997; doi:10.1186/s12915-020-00888-z)
Supplement: Supplementary file 3 — Additional file 3: Fig. S3 Coomassie-stained SDS-PAGE of proteolytic fragments of isolated Tom70 (residues 38–617) obtained by incubation with trypsin. [file 12915_2020_888_MOESM3_ESM.pdf]

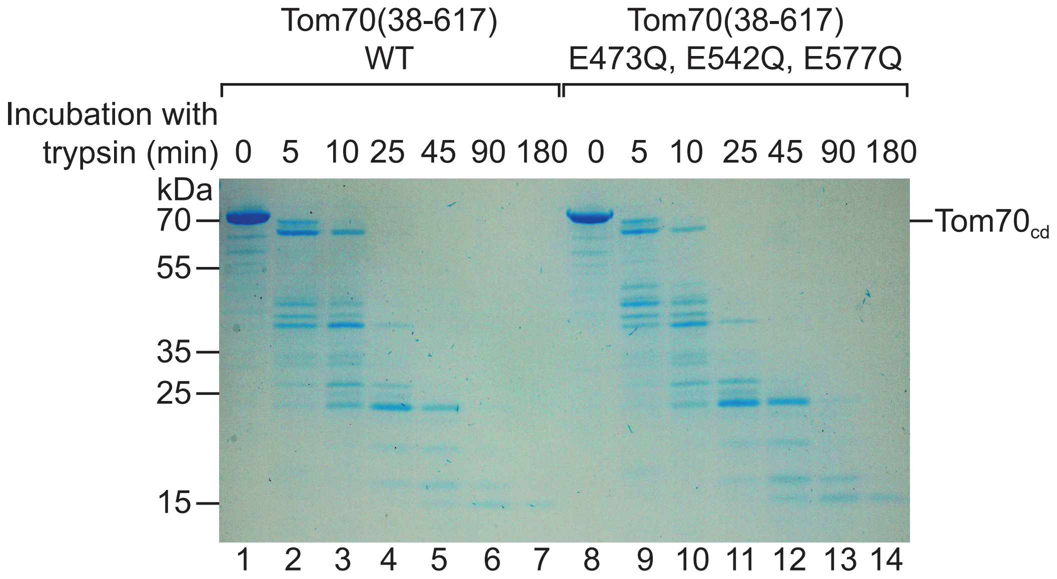


**Figure S3. SDS-PAGE and Coomassie staining of proteolytic fragments of isolated Tom70$_{cd}$.** The purified cytosolic domain of yeast Tom70 (Tom70$_{cd}$, residues 38-617) was incubated with trypsin (5 µg/ml) at 0 °C for different times as indicated. In parallel, a purified derivative was incubated containing glutamine residues in the positions of the glutamates E473, E542 and E577 (Tom70$_{\Delta Glu}$).
