# Supplementary material for: The selectivity filter of the mitochondrial protein import machinery
Source: BMC Biol. 2020 Oct 29;18:156. doi: 10.1186/s12915-020-00888-z (PMC7596997; doi:10.1186/s12915-020-00888-z)
Supplement: Supplementary file 4 — Additional file 4: Fig. S4 Data on the binding of AAC modules I, II and III to isolated Tom70 and to isolated mitochondria. [file 12915_2020_888_MOESM4_ESM.pdf]

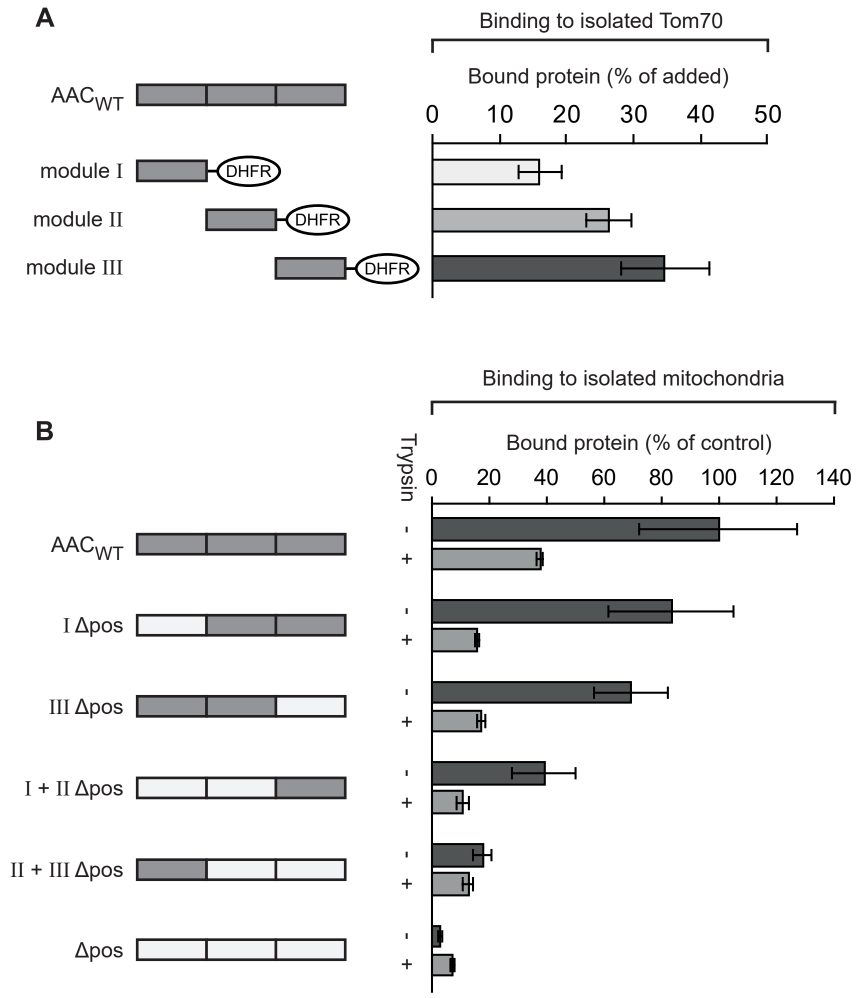

Figure S4. Binding of AAC modules I, II and III to isolated Tom70 and to mitochondria.

A, Binding of individual AAC modules to isolated $Tom70_{cd}$. [^{35}S]-labeled modules I (residues 10-100), II (residues 117-206) or III (residues 226-305) were fused to mouse dihydrofolate reductase (DHFR), synthesized in reticulocyte lysate and incubated with $Tom70_{cd}$. The assay was carried out as in the experiment of Fig. 3B. The total amounts of radiolabelled protein added per sample were set to 100%. SD, n ≥ 4. **B**, Binding to isolated mitochondria of [^{35}S]-labeled wild type AAC and of derivatives of AAC in which all positively charged residues were exchanged against glycine residues in module I (I Δpos), module III (III Δpos), modules I and II (I + II Δpos), modules II and III (II + III Δpos), or in all three modules (Δpos). As indicated, the mitochondria were pretreated with trypsin (+ Trypsin) or left untreated (- Trypsin). All samples were depleted of ATP by incubation with apyrase. Following incubation with the radiolabeled constructs, the mitochondria were reisolated, the proteins were separated by SDS-PAGE, and the relative amounts of radiolabeled proteins were determined. The average amounts of mitochondria-bound wild type AAC were set to 100% (control). SD, n = 3.
