# Supplementary material for: The selectivity filter of the mitochondrial protein import machinery
Source: BMC Biol. 2020 Oct 29;18:156. doi: 10.1186/s12915-020-00888-z (PMC7596997; doi:10.1186/s12915-020-00888-z)
Supplement: Supplementary file 5 — Additional file 5: Fig. S5 Data on the targeting of the peroxisomal protein Ant1p. [file 12915_2020_888_MOESM5_ESM.pdf]

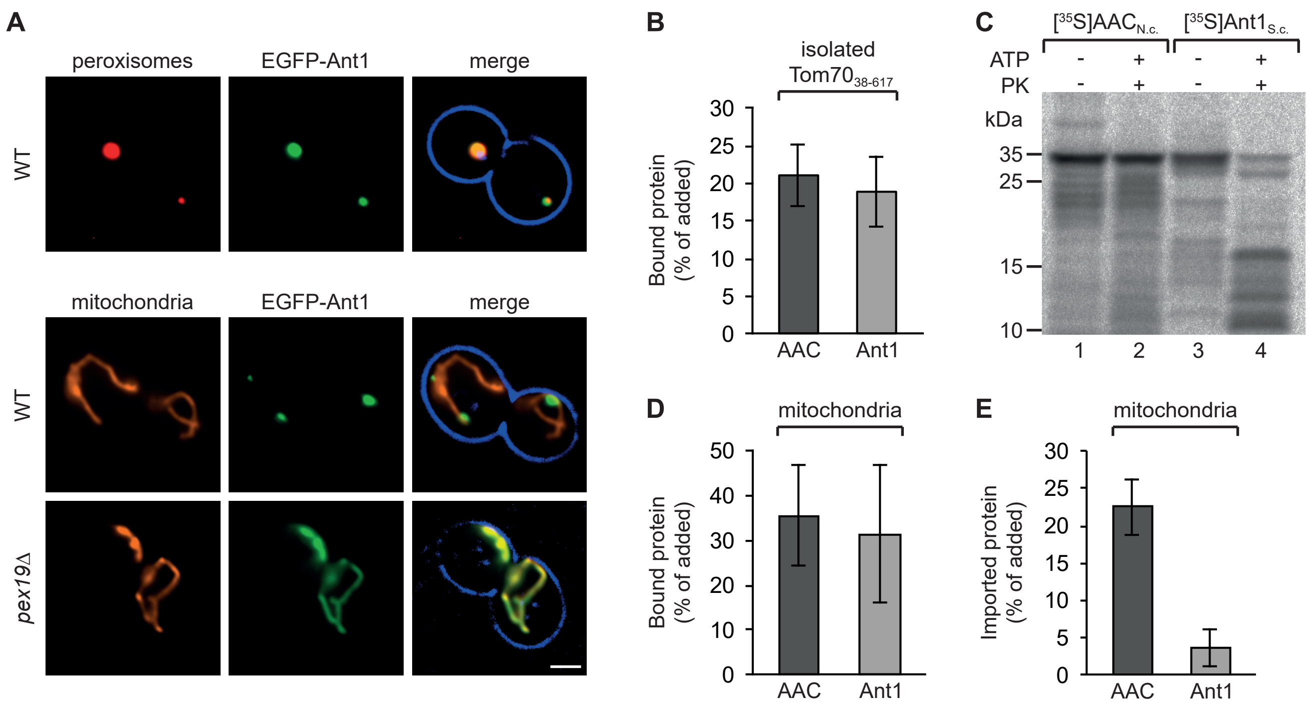

Figure S5. Targeting of Ant1p. A, Upper panel: Images of wild type yeast cells expressing Ant1p labeled by an EGFP moiety (green) and the protein mCherry-SKL (red, containing the peroxisomal targeting signal Ser-Lys-Leu). Blue outlines visualize the boundaries of the cells. Second panel: Yeast wild type cells expressing EGFP-labeled Ant1p (green) were incubated with MitoTracker (orange). Lower panel: Expression of EGFP-Ant1p in a yeast *pex19Δ* deletion strain and labeling of mitochondria with MitoTracker Orange; bar 2 µm. **B**, Binding of [^{35}S]-labeled AAC and [^{35}S]-labeled Ant1p to isolated Tom70$_{cd}$. The assay was carried out as in experiment of Fig. 3B. SD, n = 6. **C**, Import of [^{35}S]-labeled AAC (33.9 kDa) and Ant1p (36.4 kDa) into isolated yeast mitochondria. Reticulocyte lysates and mitochondria were incubated with apyrase (samples 1 and 3) or left untreated (samples 2 and 4) and then mixed and incubated at 25 °C. Samples 2 and 4 were incubated with proteinase K. The mitochondria were reisolated for subsequent analysis. **D**, Quantification. The amounts of bound radiolabeled protein as shown in (C), lanes 1 and 3, were determined in relation to the total amounts added to the samples, SD, n = 12. **E**, Mitochondrial import of [^{35}S]-labeled AAC and Ant1p in two steps: Both proteins were incubated with mitochondria in the absence of ATP (as in C). The mitochondria were then reisolated and resuspended. One half was incubated at 25 °C in the presence of 2 mM ATP and subsequently treated with proteinase K. The other half was used to determine the total amount of mitochondria-associated radiolabeled protein. The ratio of both values was calculated, SD, n = 12.

Fig. S5
